# Diversity of Marine Macro-Algicolous Endophytic Fungi and Cytotoxic Potential of *Biscogniauxia petrensis* Metabolites Against Cancer Cell Lines

**DOI:** 10.3389/fmicb.2021.650177

**Published:** 2021-06-14

**Authors:** Subhadarsini Sahoo, Kamalraj Subban, Jayabaskaran Chelliah

**Affiliations:** Department of Biochemistry, Indian Institute of Science, Bengaluru, India

**Keywords:** biodiversity, algicolous fungi, cytotoxicity, *Biscogniauxia petrensis*, secondary metabolites

## Abstract

Hypersaline environments are known to support diverse fungal species from various orders. The production of secondary metabolites is one of the strategies that fungi adopt to thrive under such extreme environments, bringing up the stress tolerance response. Some such unique secondary metabolites also exhibit clinical significance. The increasing prevalence of drug resistance in cancer therapy demands further exploration of these novel bioactive compounds as cancer therapeutics. In the present study, a total of 31 endophytic fungi harboring inside red, green, and brown marine algae have been isolated and identified. The maximum likelihood analysis and diversity indices of fungal endophytes revealed the phylogenetic relationship and species richness. The genus *Aspergillus* was found to be the dominating fungus, followed by *Cladosporium* spp. All the isolated endophytic fungal extracts were tested for their cytotoxicity against HeLa and A431 cancer cell lines. Nine isolates were further analyzed for their cytotoxic activity from the culture filtrate and mycelia extract. Among these isolates, *Biscogniauxia petrensis* showed potential cytotoxicity with CC_50_ values of 18.04 and 24.85 μg/ml against HeLa and A431 cells, respectively. Furthermore, the media and solvent extraction optimization revealed the highest cytotoxic active compounds in ethyl acetate extract from the potato dextrose yeast extract broth medium. The compound-induced cell death *via* apoptosis was 50–60 and 45% when assayed using propidium iodide-live/dead and loss of mitochondrial membrane potential assay, respectively, in HeLa cells. Four bioactive fractions (bioassay-based) were obtained and analyzed using chromatography and spectroscopy. This study reports, for the first time, the cytotoxic activity of an endophytic fungal community that was isolated from marine macro-algae in the Rameswaram coastal region of Tamil Nadu, India. In addition, *B. petrensis* is a prominent apoptotic agent, which can be used in pharmaceutical applications as a therapeutic.

## Introduction

Cancer is one of the major causes of illness and death globally (Nagai and Kim, 2017). The current cancer treatments comprise of surgery and radiation, followed by chemotherapy. The most extensively used treatment is chemotherapy; however, the routine use of chemically synthesized anticancer drugs suppresses the immune system. This necessitates the development of anticancer drugs which can control cancer progression in a better way. Moreover, the requirement for new and highly effective compounds which can provide assistance and relief in all aspects of human illnesses is ever growing. Therefore, the discovery of new drugs from natural sources has been the focus of research works (Wright, 2019). In this context, marine fungi are being considered as a new and promising source of bioactive compounds (Deshmukh et al., 2018).

It has been reported that there has been a lack of study on endophytic fungi’s applications in the pharmaceutical field (Sarasan et al., 2017). The marine macro-algae-associated endophytic fungi have the capability to produce novel secondary molecules, as they survive in special ecological niches of inexorable stress (prolonged periods of exposure to sunlight, sharp variation in moisture, large salt concentration, changing tides, abundant microorganisms, and insect herbivores) (Schulz and Boyle, 2005). Even though the relationship between host and endophyte is poorly understood, it has been observed that microbial endophytes enhance host fitness by producing bioactive compounds, which improve their survival against pathogens and environmental stresses and promote host growth. These active metabolites are the target of current research in drug discovery (Pietra, 1997; Strobel and Daisy, 2003; Debbab et al., 2011). Thus, an enormous chance to discover novel compounds from less-investigated marine endophytic fungi (Guo et al., 2008; Aly et al., 2011) and marine fungi has been largely neglected for many decades (Imhoff, 2016). Recently, endophytic fungi have been recognized as an important and novel resource of natural bioactive products, especially for their anticancer properties for therapeutic purposes (Jeewon et al., 2019).

The marine algae-associated endophytic fungi have already been reported in many countries such as Germany, China, Israel, Italy, United States, and South Africa (Nguyen et al., 2013; de Felício et al., 2015; Zhang et al., 2016). Moreover, such fungi and their bioactive compounds have been reported from many regions in India (Sarasan et al., 2020). A total of 199 different compounds isolated from marine fungi have shown considerable promise as cytotoxic agents (Deshmukh et al., 2017). A previous study also reported that 45 endophytic fungi have been isolated from the red macro-alga; among these, the ethyl acetate extracts of *Penicillium decaturense* showed cytotoxicity, with IC_50_ values of 20.93, 6.63, and 3.78 μg/ml, against cell lines SF-295, HCT-8, and HL-60, respectively. *Penicillium waksmanii* possessed IC_50_ values of 14.57, 4.38, and 11.73 μg/ml against SF-295, HCT-8, and HL-60 cell lines. Both strains displayed antibacterial activities, with minimum inhibitory concentration > 400 μg/ml (de Felício et al., 2015). In this context, it is necessary to study marine endophytes extensively for several therapeutic purposes. Several lines of evidence suggest that algae-associated endophytes are an outstanding source of bioactive metabolites from *Cephalosporium*, *Penicillium*, *Aspergillus, Strobilurus*, *Tolypocladium*, and *Chaetomium* (Gouda et al., 2016; Blunt et al., 2017; Shirley et al., 2018).

From this point of view, as part of our continuing search for novel cytotoxic compounds, it was found that crude ethyl acetate (EtOAc) extract has potent anticancer metabolites from algae-associated endophytes such as *Talaromyces purpureogenus* and *Aspergillus unguis* (AG1.2) from the coastal regions of Goa and Kerala (Kumari et al., 2018; Kamat et al., 2020). It has been reported that there is increasing pressure, due to temperature, high salinity, and environmental pollution, on the natural resources with rich marine algae biodiversity in Rameswaram (Thirunavukkarasu et al., 2012; Bhagyaraj and Kunchithapatham, 2016). Interestingly, another study reported and showed the distribution and diversity of endophytic fungi in 10 seagrasses by morphological taxonomy approaches in the Bay of Bengal waters at Rameswaram (Venkatachalam et al., 2015b). To the best of the authors’ knowledge, cytotoxicity against cancer cell lines has not yet been reported from algae-associated endophytic fungi from the Mandapam region of Tamil Nadu; chitinase and xylanase activities have been documented (Thirunavukkarasu et al., 2015; Venkatachalam et al., 2015a). Therefore, continuing the search for potent algae-associated endophytic fungi from the unexplored marine region of Rameswaram, the authors sought to isolate and identify such fungi to explore potentially cytotoxic metabolites and their apoptotic activity in cancerous cells.

## Materials and Methods

### Chemicals and Reagents Used for This Study

Sodium hypochlorite, cetyltrimethylammonium bromide (CTAB), phenol, ethidium bromide, Dulbecco’s modified eagle’s medium, and dimethyl sulfoxide (DMSO) were purchased from Sigma Aldrich. Potato dextrose agar (PDA) and potato dextrose broth (PDB) were procured from HiMedia. Streptomycin, penicillin, and MTT were obtained from SRL-Ranbaxy. The plasticware for mammalian cell cultures was purchased from Corning and TPP. Isoamyl alcohol and ammonium acetate were purchased from SDFCL (SD- Fine Chemicals). Magnesium chloride (MgCl_2_), dNTP, and Taq polymerase were purchased from Thermo Fisher Scientific, Bangalore. Fetal bovine serum and trypsin-ethylenediaminetetraacetic acid (EDTA) were procured from GIBCO-BRL. The analytical thin-layer chromatography (TLC) sheets (silica gel 60 GF254 with aluminum support) were acquired from Merck-Millipore. Ethanol was procured from Analytical Reagents. Ethyl acetate was purchased from Merck. The water used was deionized using a Millipore (Milli-Q) system.

### Collection of Marine Algae and Isolation of Endophytic Fungi

Marie fungi/microorganisms have, to a great extent, been dismissed indeed in spite of the fact that it is evaluated that more than 10,000 prominent marine fungi are less investigated in comparison to their earth-bound partners (Jones, 2011; Deshmukh et al., 2018).

In the current research scenario, algae-associated endophytic fungi (Molinski et al., 2009) are under focus. A total of 18 different marine macro-algal species were collected from the intertidal zone at four locations, namely, Pamban, Kilakarai, Thonithurai, and Seeniappa Dargah in the high-salinity area of Gulf of Mannar, Rameswaram at 9.2876° N, 79.3129° E in Tamil Nadu; these are listed in Table 1. All the collected algae were identified based on morphological characteristics by an algae expert and according to Bhagyaraj and Kunchithapatham (2016). The healthy, mature, and undamaged algae were collected and transported to the laboratory in suitable sterile containers with seawater and processed within 24 h to isolate the endophytic fungi.

**TABLE 1:** List of the collected algae from the Gulf of Mannar, Rameswaram, Tamil Nadu, India.

**S. no.**	**Algae code**	**Marine algae**	**Collection site**
**Green algae**			
1	GCSS	*Gracilaria crassa*	Thonithurai
2	HCSS	*Halimeda gracilis*	Kilakarai
3	CRSS	*Caulerpa racemosa*	Pamban
4	CSSS	*Caulerpa scafeliformis*	Seeniappa Dargah
5	CASS	*Chaetomorpha antennina*	Thonithurai
6	CTSS	*Caulerpa taxifolia*	Thonithurai
7	HMSS	*Halimeda macroloba*	Pamban
8	CPeSS	*Caulerpa peltata*	Kilakarai
9	EFSS	*Enteromorpha flexuosa*	Seeniappa Dargah
**Brown algae**			
10	PTSS	*Padina tetrastromatica*	Kilakarai
11	TCSS	*Turbinaria conoides*	Kilakarai
12	SMSS	*Sargassum myriocystum*	Pamban
13	SMaSS	*Stochospermum marginatum*	Pamban
14	DDSS	*Dictyota dichotoma*	Thonithurai
**Red algae**			
15	GCSS	*Gracilaria corticata*	Pamban
16	HFSS	*Halymenia floresia*	Seeniappa Dargah
17	ASSS	*Acanthophora spicifera*	Seeniappa Dargah
18	CPSS	*Champia parvula*	Thonithurai

All the algal samples were washed thoroughly under running tap water, and each sample (pieces of algae) was further cut into small segments of approximately 0.5 cm and rinsed three times with sterile sea water to eliminate adherent surface debris. Then, each sample was immersed in 70% ethanol for 60–120 s for surface sterilization, followed by immersion in 4% sodium hypochlorite (NaOCl) for 60 s and washing with sterile distilled water for 10 s, separately as earlier reported (Kjer et al., 2010; Suryanarayanan et al., 2010). The samples were semi-dried with sterile tissue paper and carefully placed over the surface of a petri dish containing fresh PDA medium prepared with ASW (Holler et al., 2000). Another batch of PDA medium was prepared in sterile distilled water, and all plates were supplemented with 250 mg/L of streptomycin. The petri dishes were sealed with parafilm, labeled, and stored at 25°C under 12 h of light followed by 12 h of darkness (Suryanarayanan et al., 2010) for 4–15 days. The petri dishes were observed once every day, and the endophytes grown out of the segments were further sub-cultured in new fresh PDA plates to get pure fungal isolates. These isolates were segregated based on culture characteristics such as growth, colony surface morphology, and pigmentation (Bhagyaraj and Kunchithapatham, 2016). All the isolated fungi were sub-cultured in PDA slants, allowed to grow for 7–14 days, and stored at 4°C for future use.

### Identification of Isolated Endophytic Fungi

All the isolated different morphotypes of endophytic fungi were identified by molecular techniques. To identify the fungal isolates, each fungus was cultured in PDB medium for 7 days, and genomic DNA was isolated from fresh mycelium using the phenol–chloroform–CTAB method (Moller et al., 1992). The isolated genomic DNA was quantified using NANODROP (Thermo, United States), and the quality was assessed by visualization on 0.8% agarose gel. The isolated genomic DNA was used as a template for PCR to amplify the ITS1 and ITS2 regions using the universal primers ITS1 (CTTGGTCATTTAGAGGAAGTAA) and ITS4 [CAGACTT (G/A) TA (C/T) ATGGTCCAG], respectively. The PCR reaction mixture and amplification conditions were chosen according to the description by White et al. (1990); the reaction was carried out in a thermocycler (Techne, TC-512, United Kingdom). The PCR-amplified products were examined for purity and amplicon size by visualization on 1% agarose gel. The respective PCR product was purified (Thermo Scientific GeneJET Gel Extraction Kit) and sequenced using Sanger’s method, and a similarity of ITS gene sequences search was performed using GenBank Basic Local Alignment Search Tool for nucleotide (BLASTn) (Altschul et al., 1990). Based on the search identity and taxonomic status, the fungi were identified.

### Diversity, Species Richness, and Phylogenetic Analysis of Endophytic Fungi

The colonization frequency (CF) of each fungal isolate was observed, and the percentage of colonization frequency (CF%) was calculated. Furthermore, the Menhinick’s index (I Mn), occurrence of each fungal species, and species richness for each group of algae (red, green, and brown) were calculated using the formulae given below:

$$\mathrm{CF}\%=\frac{\mathrm{Number}\mathrm{of}\mathrm{colonies}\mathrm{isolated}\mathrm{per}\mathrm{species}}{\mathrm{Number}\mathrm{of}\mathrm{segments}\mathrm{screened}}\times 100$$

$$\mathrm{Species}\mathrm{richness}=\frac{\mathrm{Number}\mathrm{of}\mathrm{species}\mathrm{obtained}\mathrm{per}\mathrm{group}\mathrm{of}\mathrm{algae}}{\mathrm{Total}\mathrm{number}\mathrm{of}\mathrm{species}\mathrm{obtained}}$$

$$I\mathrm{Mn}=\frac{S}{\sqrt{N}}$$

where *S* = number of species and *N* = total number of individuals.

In addition, diversity indices such as Simpson’s index and Shannon diversity index were calculated using the EstimateSWin910 software (Colwell and Elsensohn, 2014).

For phylogenetic analysis, similar ITS sequences of the fungal isolates were obtained from GenBank through BLASTn analysis. Furthermore, the sequences were subjected to multiple sequence alignment by the ClustalW program, and gaps were removed from the sequences. The highly relevant sequences were used for construction of the phylogenetic tree using maximum parsimony by the MEGA 6 software (Tamura et al., 2013); *Amanita muscaria* was used as an outgroup for the phylogenetic tree.

### Cultivation of Endophytic Fungi and Preparation of Culture Extract

The endophytic fungi were inoculated in the center of petri dishes containing PDA medium and incubated at 25 ± 2°C in the dark for 7 days. The pure mycelia of each fungus (fresh; 12 plugs of 9 mm) were inoculated in a 1,000-ml flask containing 300 ml of PDB and kept in the dark at 25 ± 2°C for 21 days. On completion of 21 days, the entire culture was passed through two layers of cheesecloth to separate the mycelia and the culture filtrate. The mycelia were crushed in a sterile mortar and pestle with liquid nitrogen to obtain powder. The mycelia powder and culture filtrate were mixed together in the form of a suspension (heterogeneous mixture) in which the mycelia powder was floating around freely in the culture filtrate. The internal phase (mycelia solid powder) is dispersed throughout the external phase (liquid culture filtrate) by mechanical agitation using a shaker at 200 rpm for 12 h with the double volume of suspending solvent ethyl acetate. Furthermore, ethyl acetate solvent extract was separated from the culture filtrate and filtrated to remove the mycelia fine debris. The solvent was removed from the organic extract using a rotary evaporator (IKA RV 10 digital, Sweden). The fungal metabolites were highly concentrated under speed vacuum at 35°C (LABCONCO, United States) and stored at −20°C for further experimental use.

### Cancer Cell Lines and Their Maintenance

HeLa (Human cervical adenocarcinoma), A431 (skin cancer cells), HepG2 (human liver cancer cell line), MCF-7 (breast cancer cells), and HEK (human embryonic kidney) cell lines were procured from the National Centre for Cell Science (NCCS), Pune. They were maintained in Dulbecco’s modified eagle’s medium supplemented with 10% fetal bovine serum, penicillin (100 IU ml-1), and streptomycin (100 IU ml-1) in a humidified 5% CO_2_ atmosphere at 37°C for experiments.

### Cytotoxic Activity of Fungal Extracts Against Human Cancer Cells

The cytotoxicity of fungal secondary metabolites against both HeLa, A431 cancer cell lines, and normal healthy cells (HEK) was assessed using the MTT assay (Mosmann, 1983). For the study, approximately 1 × 10^4^ cells per well were seeded in a 96-well plate and allowed to acclimatize overnight. Then, the cells were treated with fungal extract (5, 25, 50, and 100 μg/ml) prepared using 4% DMSO and filtered by syringe filters (0.22 μm) for a period of 24 h at 37°C in a CO_2_ incubator. At post-treatment, 20 μl of 5 mg/ml MTT solution was added to each well of the 96-well plate and further incubated for 3 h. After incubation, the medium was discarded, and the formazan crystals formed were dissolved by adding 200 μl of DMSO. The optical density (OD) was measured at 570 nm using a microplate reader (VersaMax^TM^ Tunable Microplate Reader, United States). The percentage of cytotoxicity exhibited by the cancer cells upon treatment with each fungal extract was evaluated using the following formula: cytotoxicity (%) = [1 – (OD of treated cells/OD of untreated cells) × 100]. Then, 50% of cytotoxic concentration CC_50_ was calculated for each fungal extract, and the selective index (SI) was analyzed using the formula: CC_50_ of normal healthy cells (HEK) / CC_50_ of cancer cells.

### Preparation of Culture Filtrate and Mycelial Extract for Cytotoxic Assay

To find out whether the cytotoxic metabolites are bound with mycelia or secreted into the culture filtrate, the ethyl acetate extract of the mycelium and the culture filtrate were tested separately for cytotoxicity on both HeLa and A431 cancer cell lines. For this purpose, the selected fungi were cultured in 1,000-ml flasks containing 300 ml of PDB medium and incubated in the dark at 25 ± 2°C for 21 days. After 21 days, the culture was harvested by filtration through two layers of cheesecloth to separate the mycelia and the culture filtrate. The mycelia were dried at 60°C overnight, and their dry weight was determined. Then, the mycelia were powdered using liquid nitrogen, and the intracellular metabolites were extracted with a 5X volume of ethyl acetate, whereas the culture filtrate was extracted using double the volume of ethyl acetate (Dhayanithy et al., 2019). The organic phase was collected by a separating funnel and evaporated to dryness using a vacuum rotary evaporator at 45°C. Each fungal dry solid residue of mycelia and culture filtrate extract was quantified and prepared to test the cytotoxicity.

### Characterization of *Biscogniauxia petrensis*

The endophytic fungal isolate *B*. *petrensis* was inoculated in petri dishes containing fresh PDA medium and incubated at 25 ± 2°C in the dark for 7 days. The mycelia pattern and conidial morphology were characterized using a phase-contrast light microscope (Zeiss AX10 Imager A2, Zeiss, Germany). Thirty to 50 conidia were studied to confirm the species level.

### Cytotoxic Potential of *B. petrensis* Metabolites Using Different Media and Solvents

A proven fact is that the media used for cultivation also plays an important role in the production of secondary metabolites by the fungus (Bode et al., 2002). Hence, in the present study, *B. petrensis* was further subjected to optimization in 11 different liquid media (Supplementary Table 1). The four agar plugs containing *B. petrensis* mycelia (9 mm) were inoculated in 500-ml flasks containing 100 ml of different media separately. All the flasks were incubated in dark condition at 25 ± 2°C for 21 days. Then, the cultures were harvested, and metabolites from the culture filtrate and the mycelia were extracted separately using ethyl acetate. Each fungal extract was assayed for cytotoxicity at a concentration of 25 μg/ml on HeLa and A431 cells using the MTT assay. Furthermore, to find out the cytotoxic effect of different organic solvent extracts of *B. petrensis*, the culture was inoculated in 1,000-ml flasks containing 300 ml of potato dextrose yeast extract broth (PDYEB) medium. The cultures were grown for 21 days and harvested. The mycelia, culture filtrate, and total culture were extracted separately using five different solvents, namely, ethyl acetate, dichloromethane, chloroform, hexane, and diethyl ether. The solvents were completely removed under reduced pressure using a rotary evaporator. The organic solvent extracts were prepared (25 μg/ml) and tested for anticancer activity against A431 and HeLa cell lines.

### Live/Dead Viability Assay

Propidium iodide (PI), a fluorescent dye, binds to DNA by intercalating between the bases and is commonly used to detect dead cells in a population, as it is not permeable in live cells. To observe the cytotoxic effect of EtOAc extracts of mycelia and culture filtrate on HeLa cells, a PI live/dead assay was performed as reported earlier (Chakravarthi et al., 2013). For this purpose, HeLa cells (2.5 × 10^4^/ml) were seeded in a 24-well culture plate and allowed to adhere overnight. Furthermore, the cells were treated with two different concentrations (25 and 50 μg/ml) of *B. petrensis* culture filtrate extract (BpCFE) and mycelial extract (BpME) prepared using 4% DMSO for 24 h. After incubation, the cells were trypsinized and centrifuged (3,000 rpm, 3 min), and the pellet was washed twice with ice-cold × 1 PBS. The untreated cells were taken as control, and paclitaxel (12 nM)-treated cells served as a positive control. The cells were then stained with PI for 30 min at 37°C and analyzed by fluorescence-activated cell sorting (FACS) in a CytoFlex flow cytometer (Beckman coulter-CytoFLEX S). The percentage of live and dead cells was calculated using the CytExpert software.

### Measurement of Mitochondrial Membrane Potential Using JC-1 Staining

The mitochondrial membrane potential test was done with JC-1 (5,5′,6,6′-tetrachloro-1,1′,3,3′-tetraethylbenzimidazolcarbocyanine iodide) staining as described earlier (Cossarizza et al., 1993). For the experiment, 5 × 10^4^ HeLa cells were seeded per well in a 24-well plate and kept overnight. Then, the cells were treated with 25 μg/ml of BpME and BpCFE for 24 h. At post-treatment, 0.2 μM of JC-1 dye was added into the untreated and treated cells. The untreated cells were considered as control, while the 2,4-DNP (1 μM)-treated cells acted as positive control. The plates were incubated under dark conditions at 37°C for 15 min. Then, the cells were harvested, washed twice with ice cold × 1 PBS, and analyzed in a FACS instrument (Beckman coulter-CytoFLEX S). The percentage of cell population was calculated using the CytExpert software.

### Cell Cycle Analysis

The cell cycle was studied by flow cytometry as described earlier (Sowmya et al., 2015). The HeLa cells were seeded (1 × 10^5^ per well) in 12-well plates treated with 25 μg/ml ethyl acetate extract of BpME and BpCFE for 12 h. Then, the cells (in the 12-well plates) were rinsed with PBS, detached with trypsin-EDTA at room temperature, and centrifuged at 3,000 rpm for 5 min. The cells were washed twice with PBS, re-suspended in 1 ml of ice-cold PBS, 0.1% Triton X-100, and 0.1 mg/ml RNase, and incubated for 3 h at 37°C. Propidium iodide (50 mg/ml) was added, and incubation was continued for 15 min. After incubation, the cell suspension was analyzed using FACS (Beckman coulter-CytoFLEX S), and the data was plotted using the CytExpert software to determine the percentage of cells in each phase of the cell cycle (G0, G1, S, and G2M).

### Chemical Analysis of Bioactive Extracts From *B. petrensis*

#### Separation of the Bioactive Extract and Determination of Cytotoxic Activity

Thin-layer chromatography was carried out using pre-coated TLC silica sheets 60 F 254. The ethyl acetates BpME and BpCFE were dissolved in methanol and spotted on the TLC sheets separately. The TLC chromatogram was developed with an optimized solvent system, namely, ethyl acetate/chloroform/methanol (80:12.5:7.5, v/v/v). The developed chromatogram was detected under white light and ultraviolet (UV) lamp at 254 and 366 nm, respectively. The *R*_*f*_ value of each band was determined, and the band characters were noted. Furthermore, preparative TLC silica gel glass plates (1 mm in thickness) were used to get the bioactive metabolites in large amounts with the same solvent system as mentioned above. All the bands were carefully scraped off from the plates with silica gel, and the compounds of each band were eluted from silica by dissolving in methanol and centrifugation at 5,000 rpm for 3 min. The elute was collected carefully without disturbing the settled silica. The eluted compounds were further purified using a silica gel column (Qiagen, RNeasy mini Column), concentrated under vacuum in a pre-weighed vial, and evaporated using a Speed Vac at 35°C (LABCONCO, United States). The yields of the compounds were noted and tested for cytotoxicity against HeLa, A431, MCF-7, HepG2, and HEK cells using the MTT assay.

#### UV Spectroscopy and High-Performance Liquid Chromatographic Analysis

The four purified bioactive fractions were processed to determine their absorption maxima by using an ultraviolet/visible (UV/vis) spectrophotometer (Shimadzu UV–vis spectrophotometer, United States). A graph of wavelength *versus* optical density was plotted to determine λ-max. Finally, the active compounds were also checked for their purity and retention time using high-performance liquid chromatography (HPLC). The purity of the bioactive compounds (C2, C5, M3, and M4) from *B. petrensis* extracts was determined using an Agilent 1120 HPLC system with a photo iodide array detector measuring absorbance at 282, 270, 273, and 266 nm, respectively. A Phenomenex Luna C18 (5 μm, 250 × 4.6 mm) column was used in combination with the isocratic elution of the mobile phase consisting of 30% acetonitrile and 70% water. The flow rate of the mobile phase was set at 0.3 ml min^–1^. The purity and retention time of the purified metabolites present in the culture filtrate and mycelial extracts were analyzed.

#### LC-ESI-MS/MS Analysis With MetFrag Tandem MS/MS Databases

Liquid chromatography with mass spectrometry (LC/MS) provides abundant information about molecular mass for the structural elucidation of compounds when tandem mass spectrometry (MS^*n*^) is applied. Therefore, the active extracts and four purified compounds were subjected to liquid chromatography–electrospray ionization–tandem mass spectrometry (LC–ESI–MS/MS) for analysis. The fungal extracts were filtered through 0.22-μm polyvinylidene fluoride filters before injecting into the column; 20 μl of each sample was injected. The analysis was performed using the Dionex Ultimate 3000 Micro LC instrument fitted with an analytical column Agilent Poroshell 120 (4.6 × 150 mm) SB-C18 and 2.7 μm particle size and a guard column. The mobile phase consisted of water/acetonitrile (75:25, v/v), and separation was performed using iso-gradient elution with a flow rate of 0.3 ml/min and column temperature of 40°C. The Rt value of the purified compound was compared with the chromatogram of an active fungal extract to confirm its presence. ESI–MS/MS was performed using the instrument ESI-Qtof (Impact HD from Bruker) in the positive mode. An acquisition range from 50 to 1,700 m/z at a spectral rate of 1 Hz was used. The LC–MS interface was used for electrospray ionization. The mass spectrometry data was analyzed using the Bruker Compass Data Analysis software (version 4.3; Bruker Daltonics, Bremen, Germany). The MetFrag1web tool (version 2.1) was used to compare the fragment patterns of fragmented ions with existing databases such as PubChem, ChemSpider, and KEGG (Tapfuma et al., 2019).

### Statistical Analysis

The statistical values were represented by the mean of three replicates and their standard deviations (mean ± SD). All the statistical analyses were done using Microsoft Excel (Redmond, WA) and the GraphPad Prism software (version 5.03).

## Results

### Identification of Marine Algae-Associated Endophytic Fungi

**FIGURE 1:**
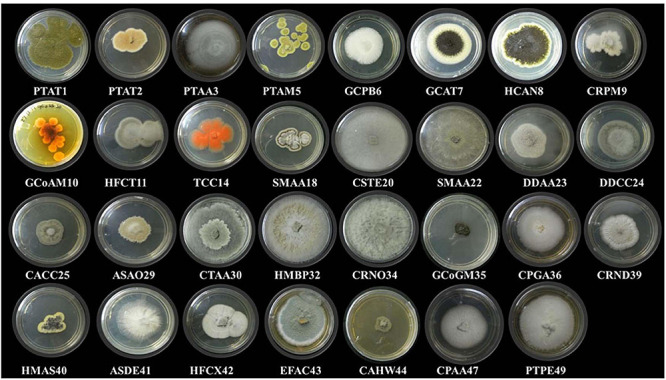
Different endophytic fungi (morpho species) isolated from marine brown, green, and red algae.

In this study, 31 different endophytic fungi were isolated from 18 marine macro-algae (Figure 1 and Supplementary Figure 1) by culturing them on a PDA medium prepared with and without artificial salt. The fungal isolates were identified based on different morphological features and molecular taxonomy using ITS gene sequences from the PCR-amplified products (Supplementary Figure 2). The obtained ITS sequences were compared with existing ITS sequences in the GenBank repository to identify the fungi. The names of the fungal isolates were obtained and confirmed according to 99.9% similarity in the GenBank database. Furthermore, the sequences, along with detailed descriptions of the endophytic fungi and their hosts, were submitted to the National Center for Biotechnology Information (NCBI) GenBank; the obtained accession numbers are mentioned in Table 2.

**TABLE 2:** Host algae and their respective endophytic fungi with CFU and NCBI accession number.

**Marine Algae**	**Gracilaria crassa**	**Halimeda crasilis**	**Caulerpa racemosa**	**Caulerpa scafeliformis**	**Chaetomorpha antenina**	**Caulerpa taxifolia**	**Halimeda macroloba**	**Caulerpa peltata**	**Enteromorpha flexuosa**	**Turbinaria conoides**	**Sargassum myriocystum**	**Stochospermum marginatum**	**Dictyota dichotama**	**Padina tetrastomatica**	**Gracilaria corticata**	**Hracilaria corticata**	**Acanthophora spicifera**	**Champia parvula**	**Total CFU**	**CFU%**	**NCBI accession number**
**Endophytic fungi**																					
**Ascomycota**																					
*Gymnascella aurantiaca* (CPGA36)	–	–	1	–	–	–	–	–	–	–	–	–	–	–	–	–	–	3	4	1.17	MH748175
*Cladosporium xantochromaticum* (HFCX42)	–	–	–	–	–	–	1	–	–	–	–	–	–	–	–	5	–	–	6	1.7	MH748179
*Amesia atrobrunnea* (SMAA18)	–	–	–	–	–	–	–	–	1	–	6	–	–	–	–	–	–	–	7	2.06	MH748152
*Amesia atrobrunnea* (PTAA3)	–	–	–	–	–	–	–	–	–	–	–	–	–	3	–	–	–	1	4	1.17	MH748153
*Periconia byssoides* (GCPB6)	–	–	–	–	–	–	2	–	–	–	–	–	–	–	7	–	–	–	9	2.65	MH748157
*Ascotricha chartarum* (EFAC43)	–	–	–	–	–	–	–	1	5	1	–	–	–	–	–	–	–	–	7	2.06	MH748180
*Nectria dematicosa* (CRND39)	–	–	–	–	–	3	–	–	–	–	–	–	1	–	–	–	–	–	4	1.17	MH748176
*Ascotricha sinuosa* (HMAS40)	–	–	–	–	–	–	6	–	–	–	–	–	–	1	–	–	1	–	8	2.35	MH748177
*Cladosporium tenuissimum* (HFCT11)	–	–	–	–	–	–	–	1	–	–	–	–	–	–	–	3			4	1.17	MH748170
*Cladosporium tenuissimum* (CACC25)	4	–	–	–	12	–	–	–	–	–	1	–	–	–	–	–	–	–	17	5.01	MH748162
*Cladosporium cladosporioides* (DDCC24)	–	–	4	–	–	–	2	–	–	–	–	–	19	–	1	–	–	–	29	8.55	MH748169
*Hortaea werneckii* (CAHW44)		1	–	–	8	–	–	1	1	–	–	–	–	–	–	–	–	–	11	3.24	MH748181
*Gliomastix murorum* (GCoGM35)	–	–	–	–	–	–	–	–	2	–	–	–	–	–	4	–	–	–	6	1.76	MH748174
**Basidiomycetes**																					
*Daldinia eschscholtzii* (ASDE41)	–	–	–	–	–	1	–	–	2	2	–	–	–	–	–	–	13		18	5.30	MH748178
**Hyphomycetes**																					
*Trichoderma erinaceum* (CSTE20)	–	–	–	7	–	–	–	–	–	–	–	–	1	–	–	–	–	1	9	2.65	MH748164
*Alternaria alternata* (DDAA23)	–	1	–	–	–	–	–	–	–	1	–	6	11	–	–	4	–	–	23	6.78	MH748168
*Aspergillus amoenus* (CPAA47)	–	–	–	–	–	–	–	4	–	–	1	–	–	–	–	–	–	–	5	1.47	MH748182
*Aspergillus tubingensis* (GCAT7)	9	–	–	–	1	–	–	–	–	2	–	–	–	–	–	–	–	–	12	3.53	MH748158
*Aspergillus terreus* (PTAT20)	–	–	–	–	–	2	–	–	–	–	–	1	–	8	–	1	–	–	12	3.53	MH748155
*Aspergillus ochraceopetaliformis* (ASAO29)	3	–	–	1	–	–	–	–	–	–	–	–	–	–		–	14		18	5.30	MH748171
*Aspergillus amstelodami* (GCoAM10)	–	–	–	–	–	–	1	–	–	–	–	–	–	–	7	–	–	2	10	2.94	MH748161
*Aspergillus amstelodami* (PTAM5)	–	–	1	–	1	–	–	–	1	–	1	–	–	9	–	1	–	1	15	4.42	MH748156
*Aspergillus niger* (HCAN8)	–	13	–	–	–	–	–	2	–	–	–	–	–	–	1	–	–	–	16	4.71	MH748159
*Aspergillus tamari* (PTAT1)	1	–	–	–	1	–	–	–	–	–	–	–	–	11	–	–	2	–	15	4.42	MH748154
*Coniochaeta sp.* (TCC14)	–	–	–	–	–	–	–	–	–	2	–	–	–	–	–	–	–	–	2	0.58	MH748163
*Biscogniauxia petrensis* (HMBP32)	–	–	–	–	–	–	7	–	1	–	–	–	–	–	–	–	–	1	9	2.65	MK073011
*Nigrospora oryzae* (CRNO34)	–	–		3	–	–	–	–	–	–	–	–	1	–	–	1	–	–	5	1.47	MH748173
*Periconia elaeidis* (PTPC49)	1	–	–	–	–	–	1	–	–	–	–	–	–	3	–	–	–	–	5	1.47	MH748183
**Coelomycetes**																					
*Phoma moricola* (CRPM9)	–	–	4	–	–	–	–	1	–	–	–	–	–	–	–	–	–	–	5	1.47	MH748160
*Aplosporella artocarpi* (CTAA30)	–	1	–	–	1	14	–	–	–	1	–	4	–	–	–	–	3	–	24	7.07	MH748172
*Aplosporella artocarpi* (SMAA22)	–	–	–	1	–	–	–	–	–	–	–	17	–	–	–	1	–	1	20	5.89	MH748167

### Diversity and Phylogenetic Study of Endophytic Fungi

Forty-seven endophytic fungal isolates were obtained from green algae, 26 from brown algae, and 23 from red algae. Interestingly, the *Aspergillus* genus was found to be dominant among the fungal isolates, exhibiting seven different species followed by three species of *Cladosporium*. Two species each of *Periconia* and *Aschotricha* were obtained, and the rest of the 13 genera were represented by only one species each (Table 2). The Shannon and Simpson’s index values were found to be 3, 2.5, and 2.58 and 15.77, 9.45, and 9.46 for green, brown, and red algae, respectively. This shows that the diversity of fungal endophytes in red and brown algae was high in comparison to that in green algae. The species richness of endophytic fungi was highest in red algae (6) but similar in brown and green algae (5 and 5.22, respectively), as shown in Supplementary Table 2. Nevertheless, it can be claimed that brown algae represent more diverse forms of fungal species as the number of host algae was less in brown (five) than in green (nine) algae. Furthermore, a phylogenetic tree has been constructed with ITS 1/5.8S rDNA/ITS 2 sequences using the MEGA6 software. This phylogenetic tree gives the evolutionary relationship between different endophytic fungi distinguished by a monophyletic group in different subclades from the outgroup (*Amanita*), as shown in Supplementary Figure 3.

### Cytotoxic Potential of Marine Endophytic Fungi

**FIGURE 2:**
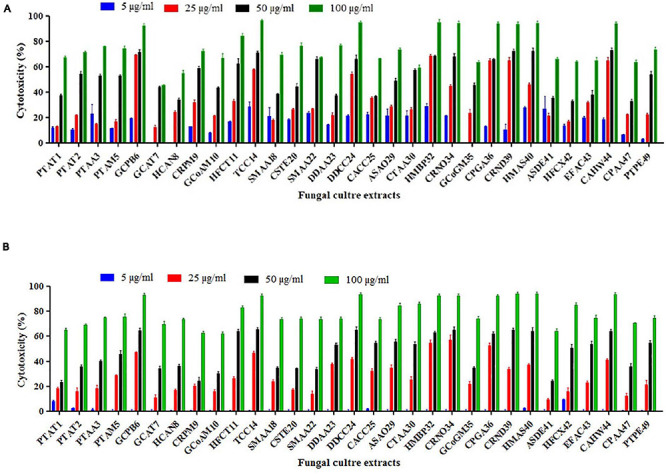
**(A,B)** *In vitro* cytotoxicity of fungal ethyl acetate extracts on human cancer cells HeLa and A431.

From the results of the MTT assay of 31 fungal extracts, the percentage of cytotoxicity was observed at a dose-dependent manner in both HeLa and A431 cells (Figures 2A,B). Though all the fungal extracts showed cytotoxicity against both cancer cells, a significant cytotoxicity was observed in the extracts of nine fungi, namely, *Periconia byssoides*, *Coniochaeta* sp., *C. cladosporioides*, *Biscogniauxia petrensis*, *Nigrospora oryzae*, *Gymnascella aurantiaca*, *Gliomastix murorum*, *Nectria dematicosa*, and *Ascotricha sinuosa*. The 50% cytotoxicity concentration (CC_50_) values for cancer cells (HeLa and A431) and normal healthy cells, along with their selectivity index, are mentioned in Supplementary Table 3. The cytotoxic effect of five fungal extracts, namely, *Amesia atrobrunnea*, *Aspergillus niger*, *Hortaea werneckii*, *Aspergillus amoenus*, and *Periconia celaeidis*, was marginal at lower doses, while it was notable at higher concentrations. The results also showed that HeLa cells were more sensitive than A431; 10–20% cell death was observed at 5 μg/ml in HeLa but not in A431.

**FIGURE 3:**
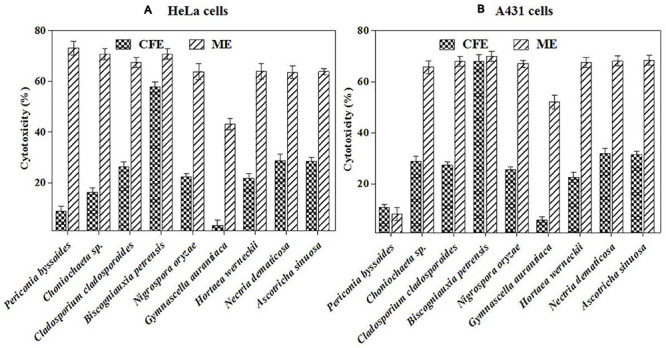
**(A,B)** *In vitro* cytotoxicity of selected fungal culture filtrates and mycelia extracts against A431 **(A)** and HeLa **(B)** cancer cell line.

Furthermore, the mycelial and culture filtrate extracts of the nine potent fungi were tested separately for their cytotoxic activities in HeLa and A431 cells. Interestingly, both the mycelial and culture filtrate extracts of *B. petrensis* showed 70% cytotoxicity on cancer cells. The mycelial extracts of six fungi showed 60% cytotoxicity, but their culture filtrate extracts were not very effective and showed less than 40% cytotoxicity. It was also observed that the extracts of *Periconia byssoides* were not effective on A431, but its mycelial extracts exhibited 70% activity on HeLa cells (Figures 3A,B). The culture filtrate extract of *G. aurantiaca* had no inhibitory effect on either HeLa or A431 cells, whereas its mycelial extract showed 50% cytotoxicity in both cells. The fungal biomass and yield of extracts were also measured; this clearly presented *B. petrensis* as the most prominent candidate compared to other fungi, with the highest biomass of 9 g (mycelial dry weight) and yields of 533 mg total extract, 733 mg mycelial extract, and 1 g culture filtrate extract obtained per liter of culture. Although the biomass and yield of extract from *P. byssoides* were of notable quantity, the cytotoxic activity was not considerable against A431 cell line. The other seven endophytic fungi showed less biomass and yield of secondary metabolites (Supplementary Figure 4).

### *Biscogniauxia petrensis* and Cytotoxic Effect of Different Media and Organic Solvent Extracts

**FIGURE 4:**
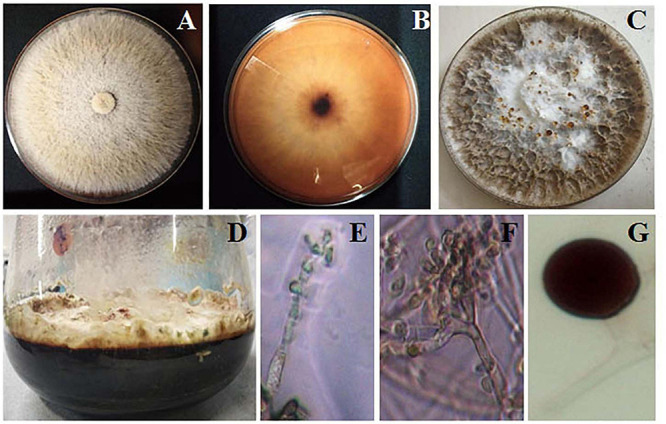
Morphological observation of *Biscogniauxia petrensis*. The colonies at 7 days of inoculation on potato dextrose agar (PDA) plate front **(A)** and rear **(B)** view, respectively. **(C)** Appearance of red droplets at 10–14 days of growth on PDA plate. **(D)** Growth of *B. petrensis* in PDYEB at 21 days of culture. **(E–G)** Light microscopy image of the *B. petrensis* spores (×40 magnification) and microscopy image of chlamydospore.

**FIGURE 5:**
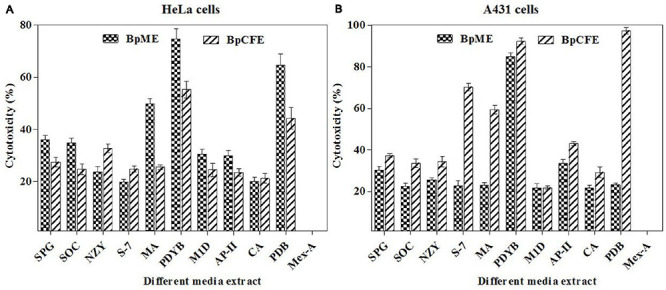
Cytotoxic effects of *B. petrensis* culture extracts grown in different media on HeLa **(A)** and A431 **(B)** at 25 μg/ml.

*Biscogniauxia petrensis* grew 80–85 mm in diameter on the PDA medium within 7 days with a cottony to wooly texture, whitish to light pink color, and with aerial mycelia. It showed dark-colored pigmentation in both solid and broth cultures (Figures 4A,B). The microscopic images showed the hyphae as brown, septate, and abundantly branched aerial mycelium. The conidiospores were yellow to light brown in color, measuring 4 to 5 μm in length, and were composed of a main axis along with one or more branches. The conidia were holoblastic, unicellular, smooth ovoid to clavate, and 5.3–7.1 μm in diameter, with an obtuse tip and acute truncated base (Figures 4C–F). The rarely seen chlamydospores were globose, 5–7 μm, dark brown, thick-walled, and attached with mycelia with a basal stalk (Figure 4G). The cytotoxicity assay of *B. petrensis* extracts obtained from 11 different culture media against HeLa and A431 cells was observed. The mycelia and culture filtrate extracts of PDYEB media showed a prominent cytotoxicity of 50–60% on both cancer cells. In the case of PDB and MB media mycelial extracts, 40–50% of cell death was observed in HeLa, while it was below 40% in A431. In addition, it was observed that the culture filtrate extracts of *B. petrensis* from S7 media was more effective on A431 (60%) in comparison to HeLa (30%). The other media extracts of *B. petrensis* showed an almost similar cytotoxicity in the range of 20–40% in HeLa and A431 cells (Figures 5A,B). In the optimization of different solvents, the ethyl acetate extracts of *B. petrensis* showed the highest cytotoxicity against both HeLa and A431 cells in the mycelial (84 and 77%), culture filtrate (74 and 83%), and total culture extracts (58 and 63%), respectively. Moreover, the mycelial extracts of dichloromethane and diethyl ether also exhibited notable cytotoxicity against HeLa cells. The mycelial chloroform extract exhibited moderate cytotoxicity on both cell lines. Ethyl acetate was found to be the most suitable out of five organic solvents, namely, hexane, diethyl ether, chloroform, ethyl acetate, and dichloromethane (Supplementary Figures 5A–C).

### Apoptotic Properties of Ethyl Acetate Extract From *B. petrensis*

#### Live/Dead Assay

**FIGURE 6:**
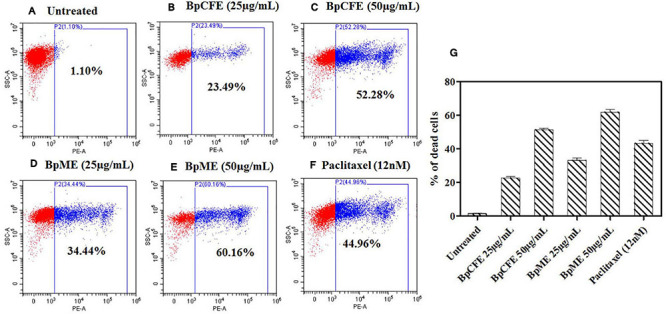
Cytotoxic effect of Bp culture extracts on HeLa cells and estimated using PI staining by FACS analysis. **(A)** Untreated **(B)** culture filtrate extract (25 μg/ml), **(C)** culture filtrate extract (50 μg/ml), **(D)** mycelial extract (25 μg/ml), **(E)** mycelial extract (50 μg/ml), **(F)** paclitaxel (12 nM), and **(G)** bar diagram representing the distribution of cell death. The data are results from three independent experiments.

The mycelial and culture filtrate ethyl acetate extracts of *B. petrensis* were tested on HeLa using the PI live/dead assay. The percentage of dead cells increased from 23 to 52% and from 34 to 60% in BpCFE- and BpME-treated cells, respectively. This indicated a dose-dependent effect of *B. petrensis* extracts when compared with the untreated (control) cells, showing 1% cytotoxicity and 45% cell death in paclitaxel-treated cells (Figure 6).

#### Effect of Fungal Extract on Mitochondrial Membrane Potential in Cancer Cells

**FIGURE 7:**
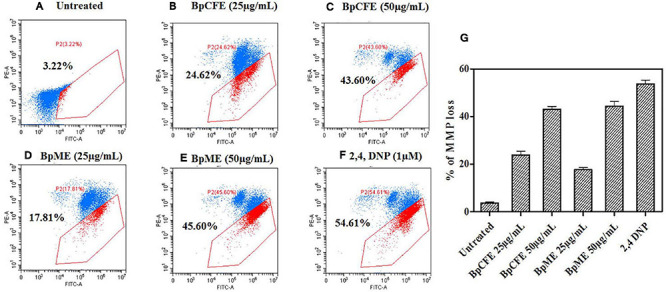
Determination of apoptosis through loss of mitochondrial membrane potential in HeLa cells induced by Bp culture extract and quantified by JC-1 monomer percentage. **(A)** Untreated **(B)** culture filtrate extract (25 μg/ml), **(C)** culture filtrate extract (50 μg/ml), **(D)** mycelial extract (25 μg/ml), **(E)** mycelial extract (50 μg/ml), **(F)** 2,4-DNP, and **(G)** statistical analysis of loss of mitochondrial membrane potential acquired in a flow cytometer. The experiments were conducted three times, and results are obtained from mean ± SD.

The MMP loss is an essential event in the mitochondrial pathway of apoptosis and can be measured using the cationic dye JC-1. The loss of MMP was observed in HeLa cells when treated with mycelial and culture filtrate EtOH extracts with two different concentrations. According to the results (Figure 7), the apoptotic cell death due to MMP loss increased from 25 to 43% in BpCFE-treated HeLa cells and from 18 to 45% in the case of BpME-treated cells at 25 and 50 μg/ml, respectively. In comparison, the untreated cells that were considered as control showed only 3.22% cell death; the 2,4-DNP-treated cells that served as positive control exhibited 54.61% cell death due to MMP loss.

#### Effect of *B. petrensis* Ethyl Acetate Extracts on Cell Cycle

**FIGURE 8:**
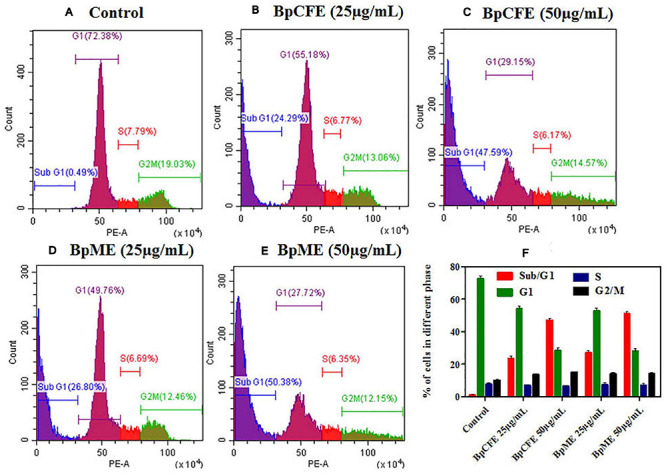
Cell cycle analysis of HeLa cells treated with Bp culture extract estimated using propidium iodide staining by flow cytometry analysis. **(A)** Untreated **(B)** culture filtrate extract (25 μg/ml), **(C)** culture filtrate extract (50 μg/ml), **(D)** mycelial extract (25 μg/ml), and **(E)** mycelial extract (50 μg/ml). **(F)** Statistical analysis showing the percentage of cell cycle in each phase. The experiments were conducted three times, and results are obtained from mean ± SD.

To examine the effect of ethyl acetate extract on cell cycle progression, the phase distribution of cells was assessed after treating the HeLa cells with BpCFE and BpME at two different concentrations for 12 h. There was a gradual increase in the percentage of cells in the sub-G1 phase from 24.29 to 47.59% in BpCFE and from 26.80 to 50.38% in BpME when treated with 25 and 50 μg/ml, respectively. The results showed a concentration-dependent accumulation of cells in the sub-G1 phase, which clearly indicates a sub-G1 phase arrest induced by the ethyl acetate extracts of *B. petrensis* (Figure 8).

#### Bioactive Compounds From *Biscogniauxia petrensis* Extracts

**FIGURE 9:**
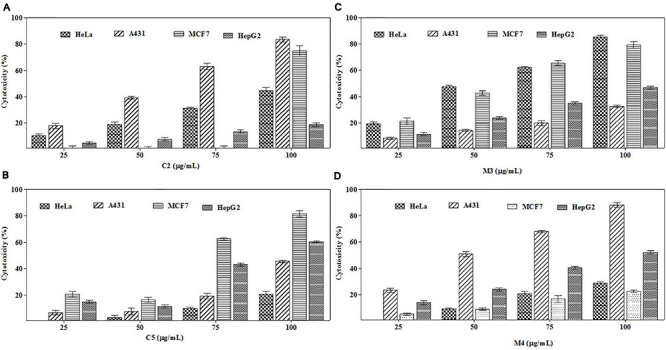
Cytotoxic effects of purified bioactive fractions from *B. petrensis* culture extracts. The cytotoxic effect was determined against HeLa, A431, MCF-7, and HepG2 cancer cell lines with different concentrations of bioactive fractions: **(A)** C2, **(B)** C5, **(C)** M3, and **(D)** M4.

To trace out the active compounds present in the crude extract, the BpME and BpCFE ethyl acetate extracts were separated by preaparative thin-layer chromatography. The *R*_*f*_ values of all the bands, along with their properties (Supplementary Figure 6), are mentioned in Supplementary Tables 4A,B. The cytotoxicity of each fraction was assessed by an MTT assay (Supplementary Figures 7A,B). Interestingly, four fractions, namely, C2 and C5 (from BpCFE) and M3 and M4 (from BpME), displayed significant cytotoxicity against all the four cancer cell lines (HeLa, A431, HepG2, and MCF7); there was no detectable effect on non-cancerous cells (HEK) (Figure 9). The results also indicated that the remaining fractions obtained from mycelia extract (16) and culture extract (12) showed a prominent cytotoxic activity against the cancer cells used in this study but also exhibited toxic effects on non-cancerous cells (HEK).

**FIGURE 10:**
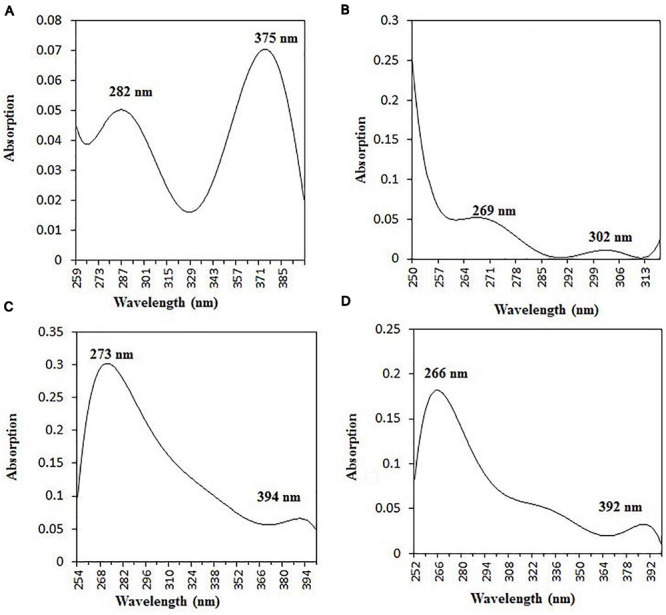
UV–vis absorbance spectra of purified bioactive fractions from *B. petrensis* culture extracts. The UV–visible spectrum of bioactive fraction showed two main absorption peaks at λ282 and λ375 for C2 **(A)**, λ269 and λ302 for C5 **(B)**, λ273 and λ394 for M3 **(C)**, and λ266 and λ392 for M4 **(D)**.

**FIGURE 11:**
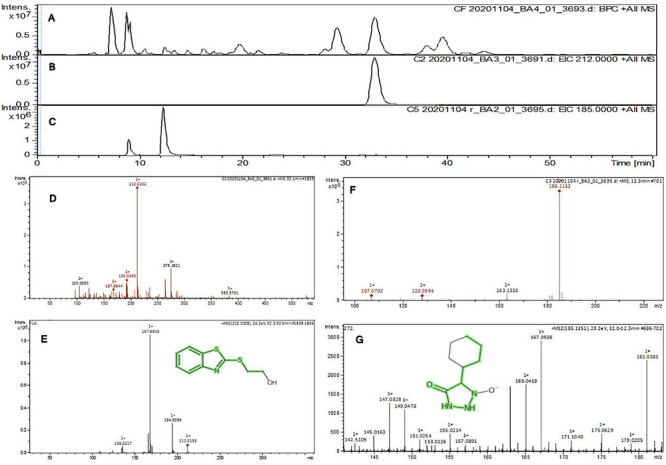
Liquid chromatogram profile from culture filtrate extract of *B. petrensis* **(A)**, liquid chromatogram profile of purified C2 fraction **(B)**, and C5 fraction **(C)**. The LC peak at Rt 33.31 and 10.13 displaying the purified bioactive fractions C2 and C5 corresponding to fungal culture filtrate extract Rt 33.31 and 10.14, respectively **(A–C)**. The mass spectrum displaying the feature m/z 212.02, 185.11 [M+H]^+^ of compounds 2-(1,3-benzothiazol-2-ylsulfanyl)ethanol and 5-cyclohexyl-1-oxido-triazolidin-4-one obtained a similar characteristic feature of molecular mass in CFE with a positive ion mode **(D–G)**.

**FIGURE 12:**
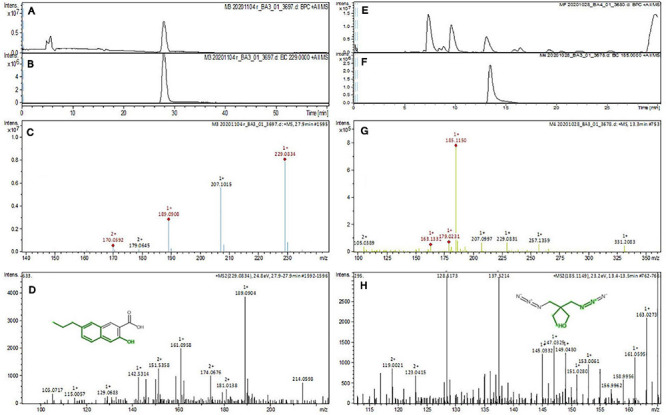
Liquid chromatogram profile from culture filtrate extract of *B. petrensis* **(A)** and liquid chromatogram profile of purified M3 fraction **(B)** and M4 fraction **(C)**. The LC peak at Rt 27.9 and 13.4 displaying the purified bioactive fractions M3 and M4 corresponding to fungal culture filtrate extract Rt 33.31 and 13.7, respectively **(A,B,E,F)**. The mass spectrum displaying the feature m/z 229.08, 185.11 [M+H]^+^ of compounds 3-hydroxy-7-propyl-naphthalene-2-carboxylic acid and 2,2-bis(azidomethyl)butan-1-ol obtained a similar characteristic feature of molecular mass in ME with a positive ion mode **(C,D,G,H)**.

The purified active principles C2, C5, M3, and M4 were tested for their purity, all of which possess *R*_*f*_ values of 0.88, 0.71, 0.66, and 0.61, respectively, with single pure spots TLC (Supplementary Figure 8). In addition, purity was further analyzed by HPLC, with C2, C5, M3, and M4 active compounds exhibiting a single peak with Rt values of 2.31, 4.74, 8.35, and 2.54, respectively (Supplementary Figure 9). The C2, C5, M3, and M4 fractions were identified using ultraviolet–visible spectrophotometry to observe the transition type experienced by the electrons of these isolated compounds. The ultraviolet spectrum of C2 showed maximum absorbance (*λ*_*max*_) at 263 and 270 nm, while for C3 it was at 222 and 244 nm. The *λ*_*max*_ values of M2 and M3 were observed to be at 222 and 244 nm and at 350–385 nm, respectively (Figure 10). The liquid chromatogram profile peak of the *B. petrensis* culture filtrate and mycelial extract displayed Rt values of 33.31, 10.13, 27.9, and 13.4 for the purified bioactive fractions C2, C5, M3, and M4, respectively, corresponding to the fungal extract Rt values of 33.31, 10.14, 33.31, and 13.7 (Figures 11, 12). The purified bioactive compounds were subjected to ESI–MS/MS to determine their molecular mass and fragmentation pattern. The bioactive metabolites C2, C5, M3, and M4 showed a mass of 212.02, 185.11, 229.08, and 185.11 m/z, respectively (Figures 11, 12). Furthermore, their MS/MS data were analyzed through the MetFrag library to find similarity matches, and these were identified as 2-(1,3-benzothiazol-2-ylsulfanyl)ethanol, 5-cyclohexyl-1-oxido-triazolidin-4-one, 3-hydroxy-7-propyl-naphthalene-2-carboxylic acid, and 2,2-bis(azidomethyl)butan-1-ol for C2, C5, M3, and M4, respectively. The information on the corresponding molecular formulae, determined based on MetFrag analysis, is mentioned in Supplementary Table 5.

## Discussion

Marine organisms comprise approximately half of the total biodiversity on earth and constitute one of the greatest sources for anticancer therapeutics (Munro et al., 1999; Faulkner, 2002). Among marine organisms, fungi are a group of biotechnologically valuable and remarkable cradle of bioactive secondary metabolites; however, they are less explored in comparison to terrestrial fungi (Deshmukh et al., 2018). A few studies have investigated marine algae and algae-associated endophytic fungal cytotoxic compounds such as gliotoxin, cytochalasin B, and demethoxyfumitremorgin (Hwang et al., 2013; Nguyen et al., 2013; Kim et al., 2017). In a very recent study, our group had documented the cytotoxic properties of marine algae-associated endophytic fungi from Kerala and Goa (Kumari et al., 2018; Kamat et al., 2020). There are many reports mentioning the diversity and bioactivity, like insecticidal, antibacterial, and antioxidant properties, of endophytic fungal secondary metabolites from the region of Rameswaram (Thirunavukkarasu et al., 2012), but no one has explored the anticancer activity. At this junction, the present study focuses on the diversity of marine endophytic fungi harbored inside macro-algae from the same region, especially highlighting the cytotoxic potential of their secondary metabolites. In the current study, 31 endophytic fungal strains were isolated from 18 different host algae that were collected from under-explored marine habitats of Gulf of Mannar at Rameswaram to find out their cytotoxic potential. It was observed that *Aspergillus* was the dominant genus of endophytic fungi among 17 genera, representing seven species (highest) out of 27 different species. The *Aspergillus* colonies were isolated from all three groups (green, brown, and red) of algae. Suryanarayanan et al. (2010) have also reported that the genus *Aspergillus* dominated the endophyte assemblage of marine algae in the coast of Tamil Nadu. Another study reported *Aspergillus* sp. as dominating all other marine-derived endophytic fungi (Sarasan et al., 2017). The results of the current study comprise all the classes of fungi such as Ascomycetes (13), Basidiomycetes (1), Hyphomycetes (14), and Coelomycetes (3).

In the preliminary screening of ethyl acetate extracts, nine fungi showed 60% notable cytotoxicity, whereas 22 fungal total extracts exhibited 30–50%. A study reported four out of 11 mangrove-associated endophytic fungi as showing cytotoxicity (cell viability, < 50%) against T47D cells (Handayani et al., 2019). The current study highlights the mycelial extract of *B*. *petrensis*, showing 70% cytotoxicity in HeLa as well as A431 cells, with the culture filtrate extracts showing 60 and 70% at 25 μg/ml. Similar results were reported by Nursid et al. (2020), indicating that mycelium extracts have stronger cytotoxicity than the culture filtrate extracts. Recently, *B. petrensis* isolated from red algae was reported to exhibit a cytotoxic activity against A549 and K562 cells from ethyl acetate extracts with CC_50_ values of 13.5 and 3.5 μg/ml, respectively (Ma et al., 2020); this confirms that potential active constituents are present in the culture extracts. However, media optimization, impact of different solvent extracts, and the apoptotic activity of the extracts and cytotoxic metabolites in *B. petrensis* have remained unexplored. The addition of one or more nutrients achieved substantial differences in the production of secondary metabolites (Frisvad, 2012; VanderMolen et al., 2013). This present study reports the production of cytotoxic secondary metabolites with nutrient availability by using different media. The PDYEB medium culture extract showed significant cytotoxicity, followed by the PDB, M1D, and S7 media culture extracts. In the past, the production of penicillin was seen to increase when the culture media were optimized from the *Penicillium* species (Dayalan et al., 2011). Various organic polar and non-polar solvents have been used to extract the bioactive compounds from microorganisms (de Felício et al., 2015; Ahsan et al., 2017). In the present study, the optimization of organic solvents for the extraction of cytotoxic secondary metabolites was studied, which enhanced the cytotoxic properties of ethyl acetate extracts. Similar results have been well established from studies of marine algae-associated endophytic fungus, which possess a cytotoxic activity (de Felício et al., 2015). A study showed the apoptotic activity of demethoxyfumitremorgin, which was isolated from a marine algae-associated fungus (Kim et al., 2017). Unlike the study by Ma et al. (2020), the present investigation shows (for the first time) that the *B. petrensis* fungal extract induces apoptosis (PI live/dead assay, loss of mitochondrial membrane potential, and cell cycle analysis), resulting in dose-dependent cell death in HeLa cells. The measurement of MMP loss by JC-1 indicated a high percentage of cell death at high concentrations of fungal extract. Similar observations were reported from macro-algae-associated endophytic fungi (Kumari et al., 2018; Kamat et al., 2020; Sajna et al., 2020).

The flow cytometry analysis revealed a sub-G1 phase arrest in HeLa cells after treatment with mycelia and culture filtrate extracts. Arora et al. (2016) have also reported fungal extract-induced sub-G1 phase arrest. Interestingly, anticancer agents (linalool and subamolide E) also induce cell death by leading to the activation of DNA damage checkpoints and sub-G1 phase cell cycle arrest (Wang et al., 2011; Sun et al., 2015). The fungal secondary metabolic profiling remains uncharacterized to recognize the bioactive compounds contributing to the cytotoxic and apoptotic activities exhibited by BpME and BpCE from *B. petrensis.* Forty-two fractions were separated from the culture filtrate and mycelial extract by preparative TLC and tested on cancer cell lines HeLa, A431, MCF-7, and HepG2 and non-cancer HEK cells by the MTT assay. All the fractions were found to inhibit the proliferation of cancer cells. Four fractions, namely, C2, C5, M3, and M4, exhibited significant cytotoxicity against all cancer cells; compared to the other fractions, they did not show a cytotoxic activity in non-cancerous HEK. A previous study showed that four preparative TLC fractions of the *Aspergillus ochraceus* extract inhibit growth in HeLa cells (Nadumane et al., 2013). In the current study, results of the MTT assay led the authors to isolate four compounds, namely, C1 and C2 (from BpCFE) and M2 and M3 (from BpME), with significant cytotoxicity; these were further characterized by spectrometry. The fractions C2, C5, M3, and M4 displayed single spots with characteristics of light orange, yellow, dark blue, and dark orange colors under a UV 365 lamp with *R*_*f*_ values of 0.88, 0.70, 0.71, and 0.66, respectively. The highest cytotoxicity was exhibited by the fraction M3 with CC_50_ value of 65 μg/ml, followed by C2, C5, and M4. The pure compound in the fractions C2, C5, M3, and M4 was soluble in methanol and had an UV λ-maximum at 375, 269, 273, and 266 nm, respectively. This guided the authors to select these four compounds for further studies. The mass and preliminary structures of these compounds were interpreted from the database using the m/z ratio obtained from LC–ESI–MS/MS (Tapfuma et al., 2019). From the matches with a score value of 1 (MetFrag), the names were confirmed as 2-(1,3-benzothiazol-2-ylsulfanyl)ethanol, 5-cyclohexyl-1-oxido-triazolidin-4-one, 3-hydroxy-7-propyl-naphthalene-2-carboxylic acid, and 2,2-bis(azidomethyl)butan-1-ol for C2, C5, M,3 and M4, respectively. Since the purified bioactive compounds from these fungal organic extracts exhibited the best cytotoxic activities, there is a promising use of these agents in cancer therapeutics.

## Conclusion

The rate of cancer occurrence has increased, with serious side effects due to chemotherapy and multidrug resistance. This has led to the search for novel and effective anticancer molecules from endophytic fungi. In this study, the authors have reported 31 marine algae-associated endophytic fungi from Rameswaram, India, for the first time, that show a cytotoxic activity against cancer cell lines. Among the fungi, *B. petrensis* extract exhibited significant cytotoxic and apoptotic effects. This is the first report of *B. petrensis* for its growth and media optimization to enhance cytotoxicity and apoptotic effects with loss of mitochondrial membrane potential. In the future, the purified cytotoxic compounds will be characterized with detailed structures, mode of action in cancer cells, and mouse models.

## Data Availability Statement

The datasets presented in this study can be found in online repositories. The names of the repository/repositories and accession number(s) can be found below: https://www.ncbi.nlm.nih.gov/, MH748175, MH748179, MH748152, MH748153, MH748157, MH748180, MH748176, MH748177, MH748170, MH748162, MH748169, MH748181, MH748174, MH748178, MH748164, MH748168, MH748182, MH748158, MH748155, MH748171, MH748161, MH748156, MH748159, MH748154, MH748163, MK073011, MH748173, MH748183, MH748160, MH748172, and MH748167.

## Author Contributions

JC conceived the idea. SS and KS did the sampling, designed and performed the experiments, analyzed the data, and wrote the manuscript. All the authors reviewed the manuscript.

## Conflict of Interest

The authors declare that the research was conducted in the absence of any commercial or financial relationships that could be construed as a potential conflict of interest.

## Supplementary Material

Supplementary Figure 1 Marine algae: green (GCSS, HCSS, CRSS, CSSS, CASS, CTSS, HMSS, CPeSS, and EFSS), brown (PTSS, TCSS, SMSS, SMaSS, DDSS), and red (GCSS, HFSS, ASSS, S) collected from four different coastal regions of Rameswaram, Tamil Nadu, India.

Supplementary Figure 2 PCR product of fungal ITS 1 and ITS 2 regions on 1% agarose gel.

Supplementary Figure 3 Phylogenetic tree of endophytic fungi obtained from marine macro-algae based on ITS regions. The phylogenetic tree was constructed using the maximum parsimony method. A bootstrap value of 100% showed that each genus was distinguished by a monophyletic group in different subclades from the outgroup.

Supplementary Figure 4 **(A)** Fungal extract yields from mycelia, culture filtrate, and total culture of the nine potent fungi. **(B)** Fungal biomass (mycelial dry weight) of the nine potent fungi.

Supplementary Figure 5 Cytotoxic effects of different solvents, namely, ethyl acetate, dichloromethane, chloroform, hexane, and diethyl ether extracts of *B. petrensis* grown in PDYEB medium. **(A)** Total extract, **(B)** culture filtrate extract, and **(C)** mycelial extract on HeLa and A431 cells at 25 μg/ml.

Supplementary Figure 6 Thin-layer chromatography (TLC) profile of *B. petrensis* mycelia (1) and culture filtrate (2) extracts. **(A)** Analytical TLC plate (sample loaded, 100 μg) and **(B)** preparative TLC plate (sample loaded, 1.6 mg).

Supplementary Figure 7 **(A)** Cytotoxic effects of purified metabolites of BpME on different human cancer cell lines. **(B)** Cytotoxic effects of purified metabolites of BpCFE on different human cancer cell lines.

Supplementary Figure 8 Thin-layer chromatography profile of purified active principles M3, M4 and C2, C5 (sample loaded, 10 μg) obtained from BpME and BpCFE, respectively.

Supplementary Figure 9 **(A)** High-performance liquid chromatography (HPLC) profile of M3 and M4 compounds in comparison with mycelial extract. **(B)** HPLC profile of C2 and C5 compounds in comparison with culture filtrate extract.

Supplementary Table 1 Composition of different media used in this study (all media mentioned below are without agar served as broth media).

Supplementary Table 2 Species richness and diversity indices of marine endophytic fungi obtained from green, brown, and red algae.

Supplementary Table 3 The 50% cytotoxic concentration (CC_50_) value of marine endophytic fungal extract against cancer cell lines HeLa, A431, and healthy cells (HEK).

Supplementary Table 4 **(A)** Thin-layer chromatography band character and retention factor (*R*_*f*_) value of the mycelia extract of *B. petrensis*. **(B)** Thin-layer chromatography band character and retention factor (*R*_*f*_) value of the culture filtrate extract of *B. petrensis*.

Supplementary Table 5 The putative identifications of bioactive compounds compared with direct Mass-based search selected peaks in MetFrag data base.
