# Supplementary material for: Diversity of Marine Macro-Algicolous Endophytic Fungi and Cytotoxic Potential of Biscogniauxia petrensis Metabolites Against Cancer Cell Lines
Source: Front Microbiol. 2021 Jun 14;12:650177. doi: 10.3389/fmicb.2021.650177 (PMC8236939; doi:10.3389/fmicb.2021.650177)
Supplement: Supplementary Figure 1 — Marine algae: green (GCSS, HCSS, CRSS, CSSS, CASS, CTSS, HMSS, CPeSS, and EFSS), brown (PTSS, TCSS, SMSS, SMaSS, DDSS), and red (GCSS, HFSS, ASSS, S) collected from four different coastal regions of Rameswaram, Tamil Nadu, India. [file Data_Sheet_1.pdf]

Supplementary information

Diversity of Marine Macro-algicolous Endophytic Fungi and Cytotoxic Potential of *Biscogniauxia petrensis* Metabolites Against Cancer Cell Lines

Subhadarsini Sahoo[1], Kamalraj Subban[1] and Jayabaskaran Chelliah[1*]

[1] Department of Biochemistry, Indian Institute of Science, Bangalore-560 012, India.

* Corresponding author.
E-mail address: cjb@iisc.ac.in (C. Jayabaskaran)

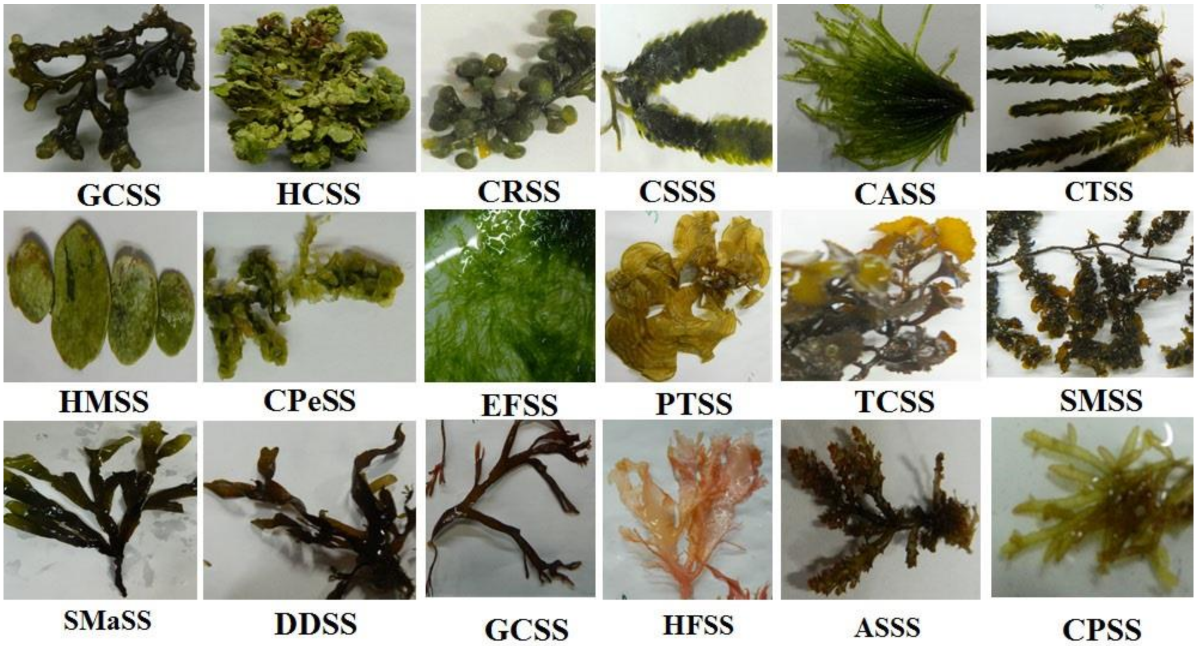

Figure S1. Marine algae green (GCSS, HCSS, CRSS, CSSS, CASS, CTSS, HMSS, CPeSS, EFSS) brown (PTSS, TCSS, SMSS, SMaSS, DDSS), red (GCSS, HFSS, ASSS, CPSS) collected from at four different costal region of Rameswaram, Tamil Nadu, India.

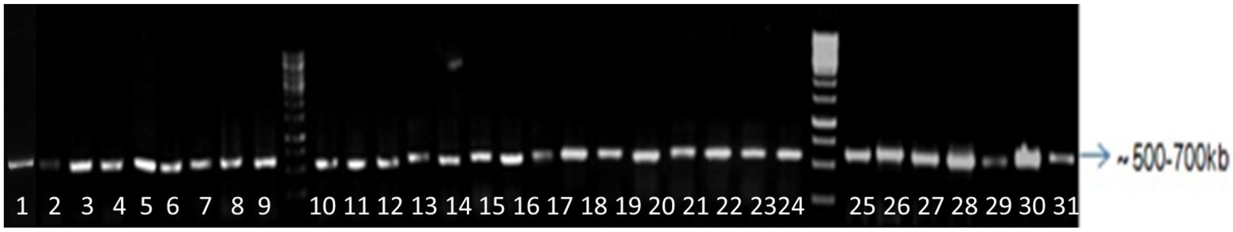

Figure S2. PCR product of fungal ITS 1 and ITS 2 region on 1% agarose gel

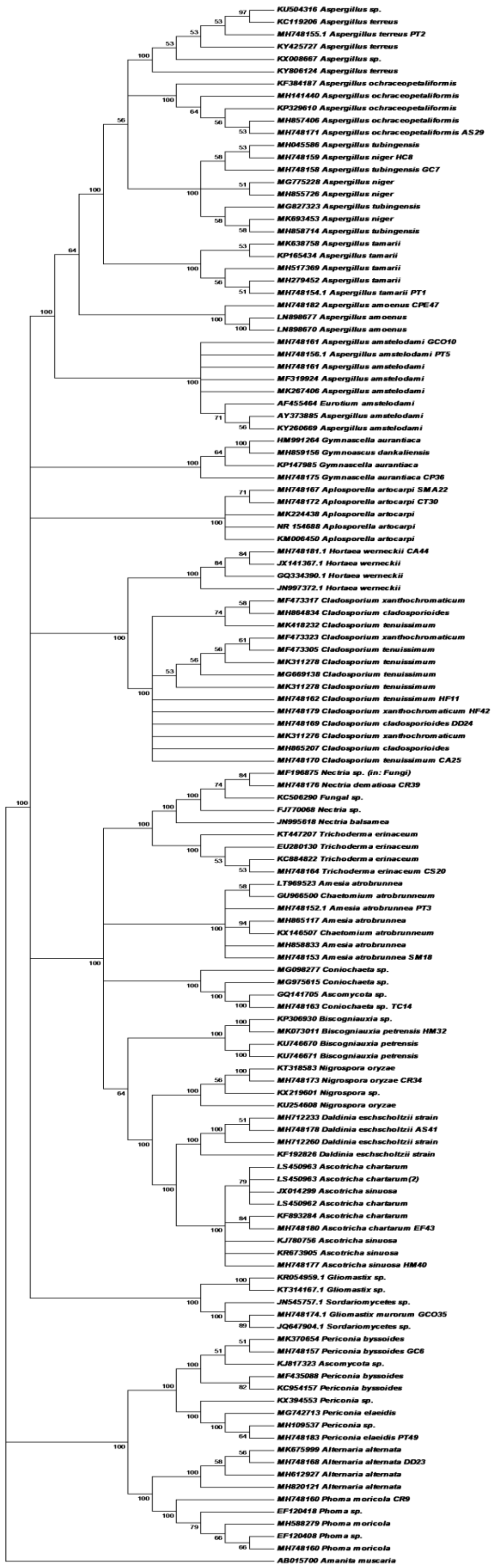
KU504316 Aspergillus sp.
KC119206 Aspergillus terreus
MH748155.1 Aspergillus terreus PT2
KY425727 Aspergillus terreus
KX008667 Aspergillus sp.
KY806124 Aspergillus terreus
KF384187 Aspergillus ochraceopetaliformis
MH141440 Aspergillus ochraceopetaliformis
KP329610 Aspergillus ochraceopetaliformis
MH857406 Aspergillus ochraceopetaliformis
MH748171 Aspergillus ochraceopetaliformis AS29
MH045586 Aspergillus tubingensis
MH748159 Aspergillus niger HC8
MH748158 Aspergillus tubingensis GC7
MG775228 Aspergillus niger
MH855726 Aspergillus niger
MG827323 Aspergillus tubingensis
MK693453 Aspergillus niger
MH858714 Aspergillus tubingensis
MK638758 Aspergillus tamarii
KP165434 Aspergillus tamarii
MH517369 Aspergillus tamarii
MH279452 Aspergillus tamarii
MH748154.1 Aspergillus tamarii PT1
MH748182 Aspergillus amoenus CPE47
LN898677 Aspergillus amoenus
LN898670 Aspergillus amoenus
MH748161 Aspergillus amstelodami GCO10
MH748156.1 Aspergillus amstelodami PT5
MH748161 Aspergillus amstelodami
MF319924 Aspergillus amstelodami
MK267406 Aspergillus amstelodami
AF455464 Eurotium amstelodami
AY373885 Aspergillus amstelodami
KY260669 Aspergillus amstelodami
HM991264 Gymnascella aurantiaca
MH859156 Gymnoascus dankaliensis
KP147985 Gymnascella aurantiaca
MH748175 Gymnascella aurantiaca CP36
MH748167 Aplosporella artocarpi SMA22
MH748172 Aplosporella artocarpi CT30
MK224438 Aplosporella artocarpi
NR 154688 Aplosporella artocarpi
KM006450 Aplosporella artocarpi
MH748181.1 Hortaea werneckii CA44
JX141367.1 Hortaea werneckii
GQ334390.1 Hortaea werneckii
JN997372.1 Hortaea werneckii
MF473317 Cladosporium xanthochromaticum
MH864834 Cladosporium cladosporioides
MK418232 Cladosporium tenuissimum
MF473323 Cladosporium xanthochromaticum
MF473305 Cladosporium tenuissimum
MK311278 Cladosporium tenuissimum
MG669138 Cladosporium tenuissimum
MK311278 Cladosporium tenuissimum
MH748162 Cladosporium tenuissimum HF11
MH748179 Cladosporium xanthochromaticum HF42
MH748169 Cladosporium cladosporioides DD24
MK311276 Cladosporium xanthochromaticum
MH865207 Cladosporium cladosporioides
MH748170 Cladosporium tenuissimum CA25
MF196875 Nectria sp. (in: Fungi)
MH748176 Nectria dematiosa CR39
KC506290 Fungal sp.
FJ770068 Nectria sp.
JN995618 Nectria balsamea
KT447207 Trichoderma erinaceum
EU280130 Trichoderma erinaceum
KC884822 Trichoderma erinaceum
MH748164 Trichoderma erinaceum CS20
LT969523 Amesia atrobrunnea
GU966500 Chaetomium atrobrunneum
MH748152.1 Amesia atrobrunnea PT3
MH865117 Amesia atrobrunnea
KX146507 Chaetomium atrobrunneum
MH858833 Amesia atrobrunnea
MH748153 Amesia atrobrunnea SM18
MG098277 Coniochaeta sp.
MG975615 Coniochaeta sp.
GQ141705 Ascomycota sp.
MH748163 Coniochaeta sp. TC14
KP306930 Biscogniauxia sp.
MK073011 Biscogniauxia petrensis HM32
KU746670 Biscogniauxia petrensis
KU746671 Biscogniauxia petrensis
KT318583 Nigrospora oryzae
MH748173 Nigrospora oryzae CR34
KX219601 Nigrospora sp.
KU254608 Nigrospora oryzae
MH712233 Daldinia eschscholtzii strain
MH748178 Daldinia eschscholtzii AS41
MH712260 Daldinia eschscholtzii strain
KF192826 Daldinia eschscholtzii strain
LS450963 Ascotricha chartarum
LS450963 Ascotricha chartarum(2)
JX014299 Ascotricha sinuosa
LS450962 Ascotricha chartarum
KF893284 Ascotricha chartarum
MH748180 Ascotricha chartarum EF43
KJ780756 Ascotricha sinuosa
KR673905 Ascotricha sinuosa
MH748177 Ascotricha sinuosa HM40
KR054959.1 Gliomastix sp.
KT314167.1 Gliomastix sp.
JN545757.1 Sordariomycetes sp.
MH748174.1 Gliomastix murorum GCO35
JQ647904.1 Sordariomycetes sp.
MK370654 Periconia byssoides
MH748157 Periconia byssoides GC6
KJ817323 Ascomycota sp.
MF435088 Periconia byssoides
KC954157 Periconia byssoides
KX394553 Periconia sp.
MG742713 Periconia elaeidis
MH109537 Periconia sp.
MH748183 Periconia elaeidis PT49
MK675999 Alternaria alternata
MH748168 Alternaria alternata DD23
MH612927 Alternaria alternata
MH820121 Alternaria alternata
MH748160 Phoma moricola CR9
EF120418 Phoma sp.
MH588279 Phoma moricola
EF120408 Phoma sp.
MH748160 Phoma moricola
AB015700 Amanita muscaria

Figure S3. Phylogenetic tree of endophytic fungi obtained from marine macro-algae based on ITS regions. The phylogenetic was constructed using maximum parsimony method. 100 % bootstrap value showed each genera were distinguished by monophyletic group in different subclades from outgroup.

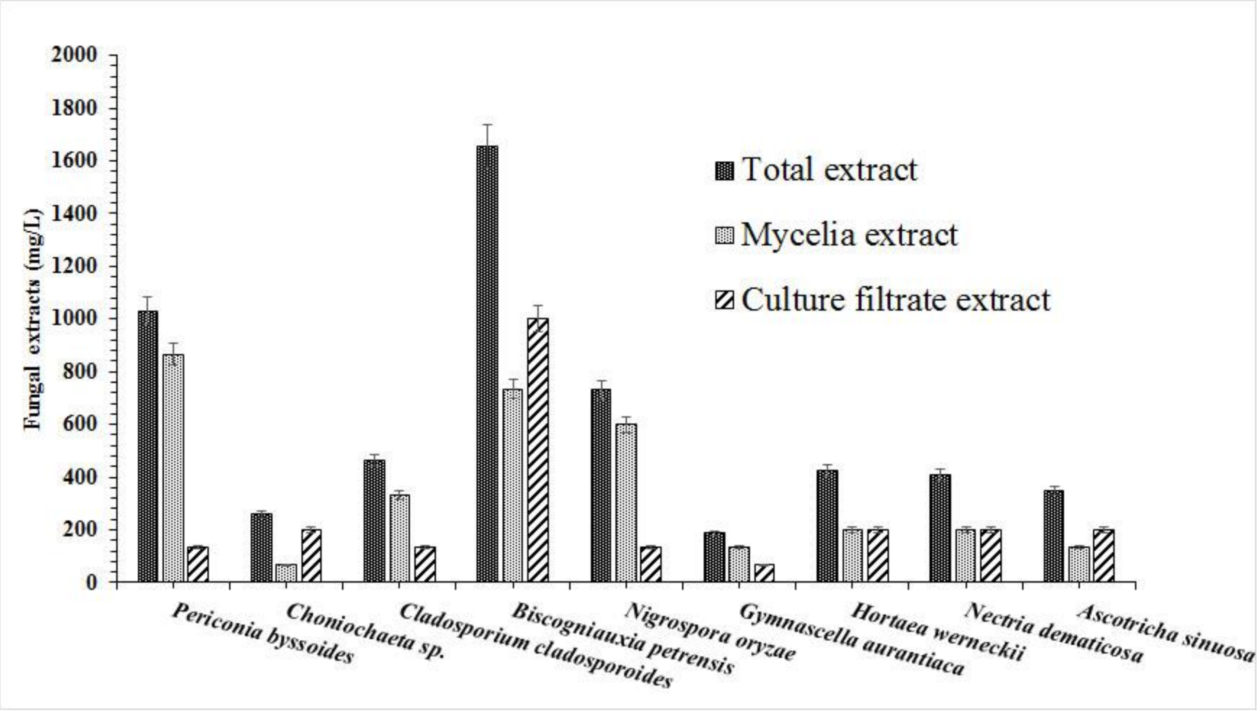

Figure S4A. Fungal yield of extracts of the nine potent fungi.

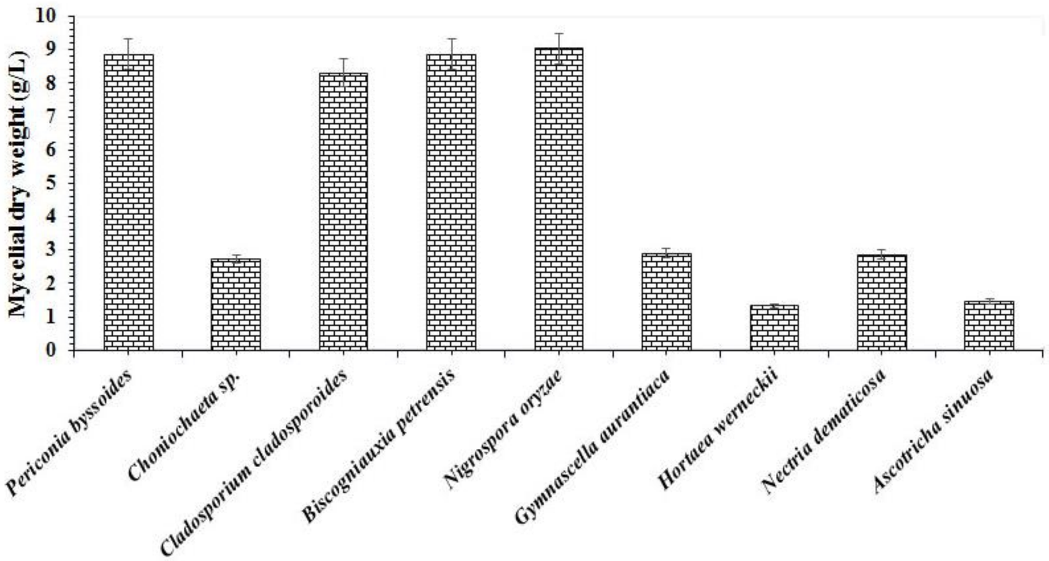

Figure S4B. Fungal biomass (mycelial dry weight) of the nine potent fungi.

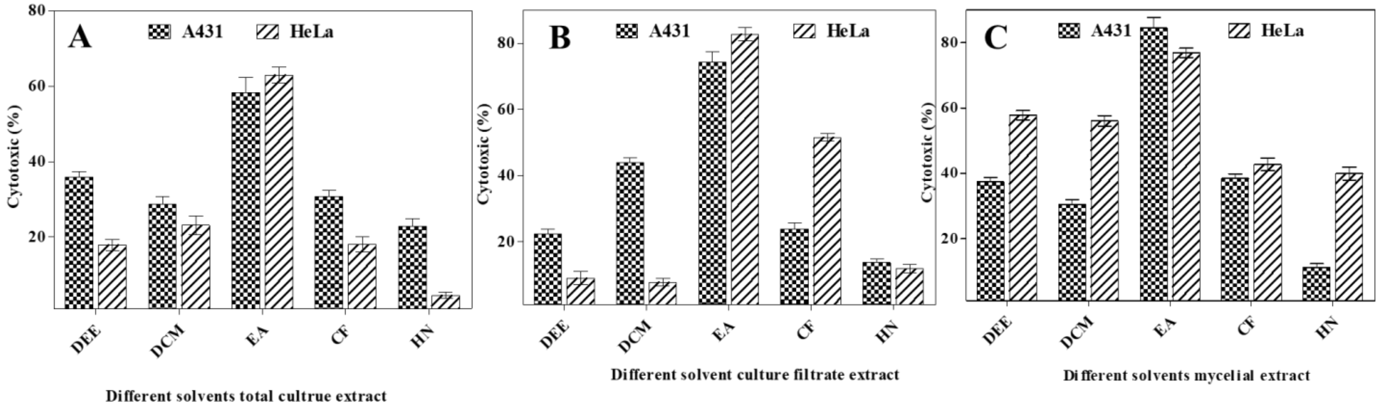

Figure S5. Cytotoxic effects of different solvent namely ethyl acetate (EA), dichloromethane (DCM), chloroform (CF), hexane (HN) and diethyl ether (DEE) extracts of *B. petrensis* grown in PDYEB medium (A) total extract, (B) culture filtrate extract and (C) mycelial extract on HeLa and A431 cells at 25µg/ml

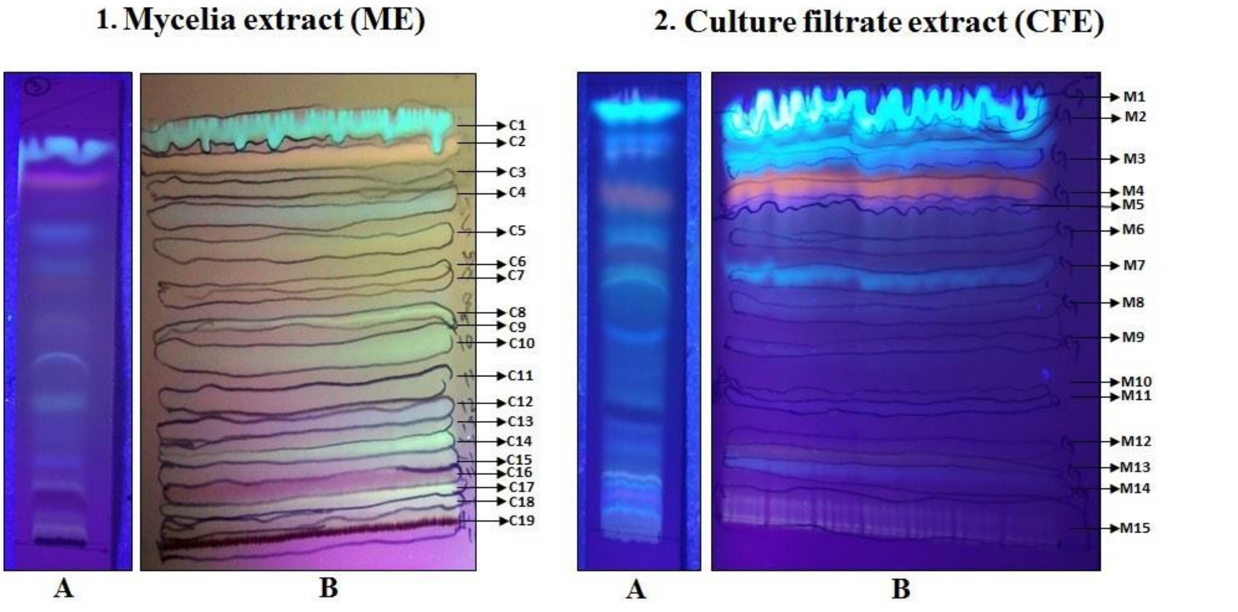

Figure S6. TLC profile of *B. petrensis* mycelia (1) and culture filtrate (2) extracts. A; analytical TLC plate (sample loaded 50 µg) and B; Preparative TLC plate (sample loaded 1.6 mg)

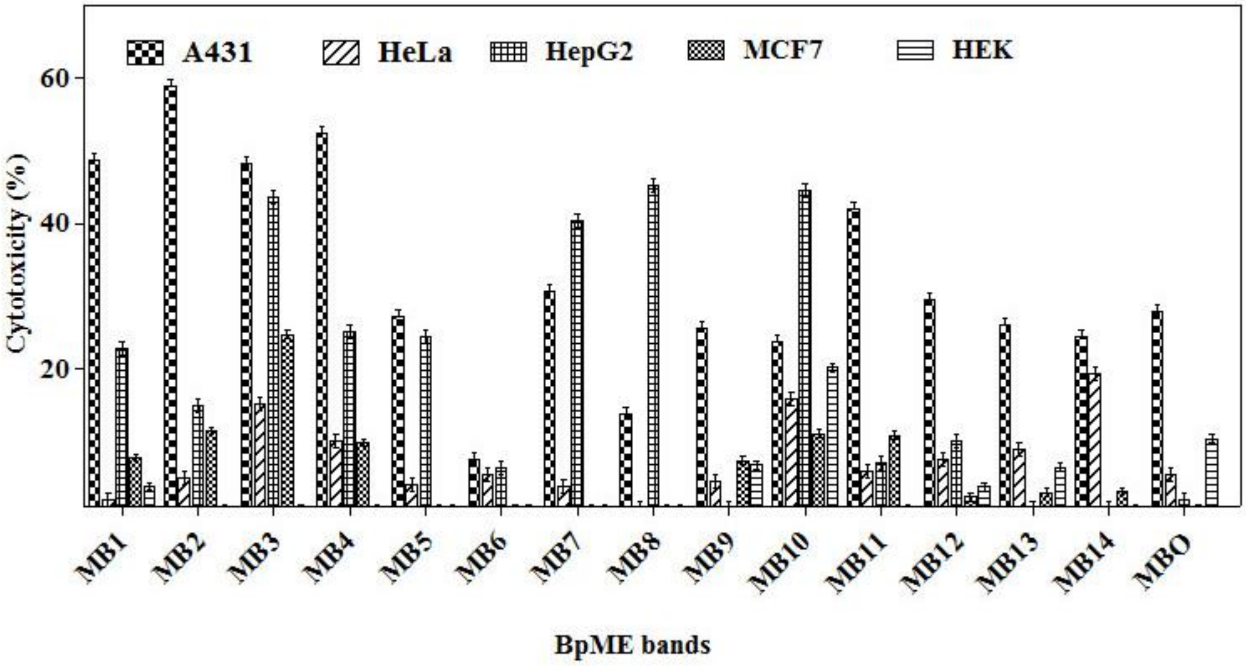

Figure S7A. Cytotoxic effects of purified metabolites of BpME on different human cancer cell lines.

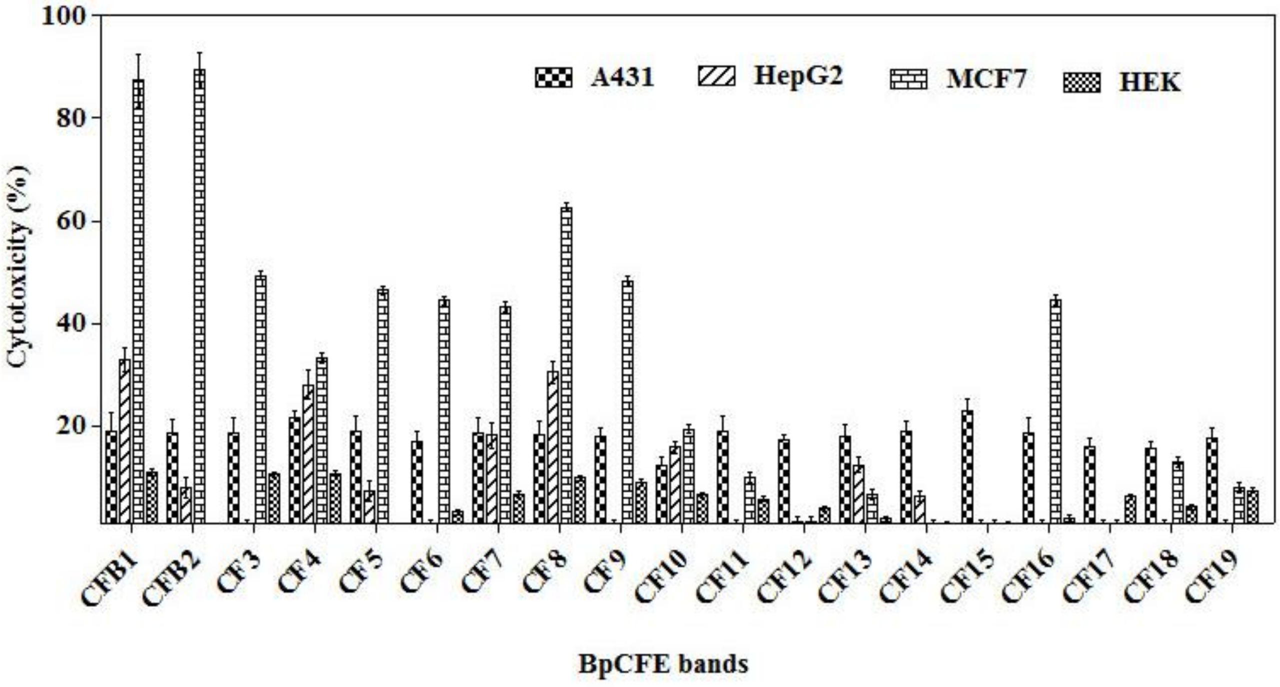

Figure S7B. Cytotoxic effects of purified metabolites of BpCFE on different human cancer cell lines.

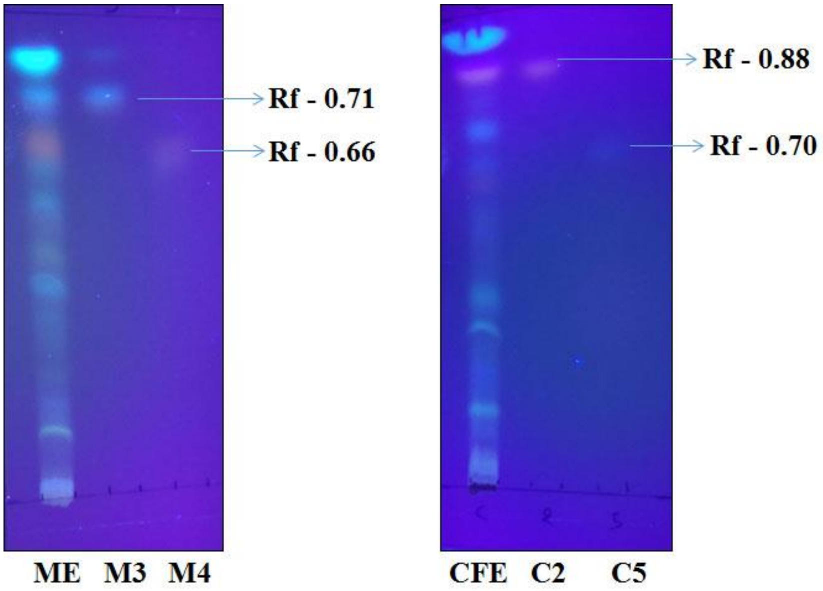

Figure S8. TLC profile of purified active principles M3, M4 (from BpME), C2 and C5 (from BpCFE).

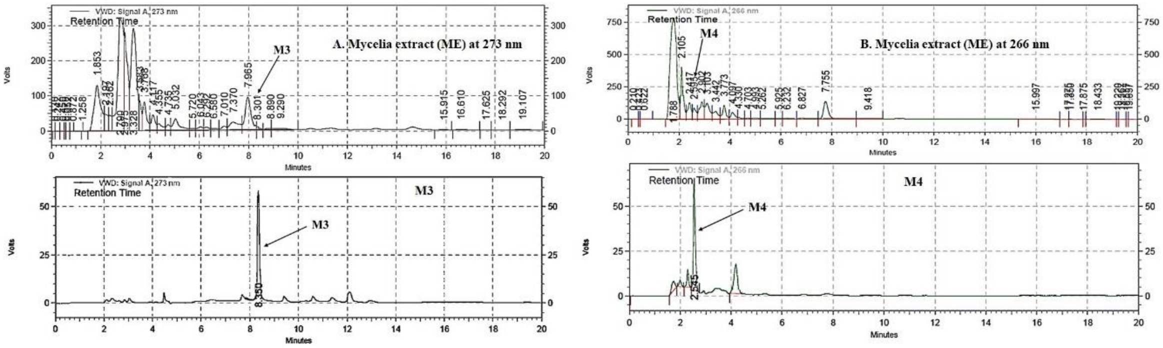

Figure S9A. HPLC profile of M3 and M5 compounds in comparison with mycelial extract.

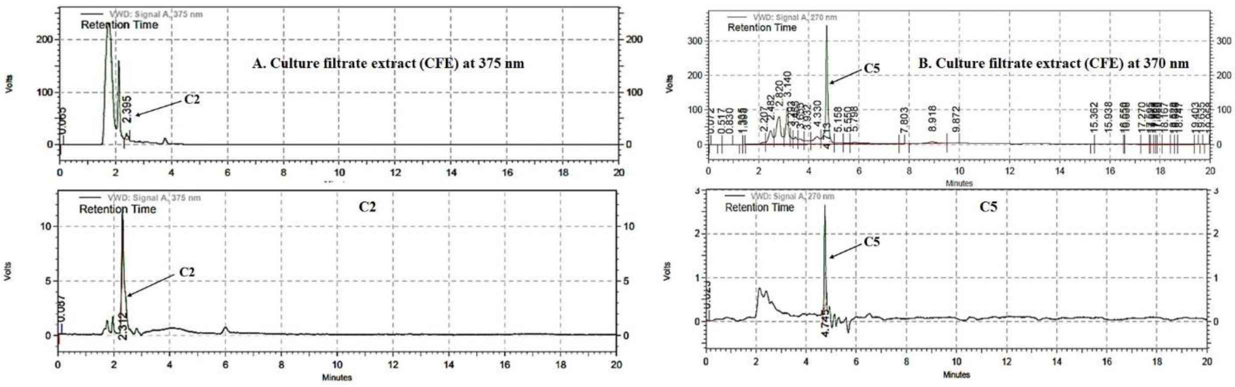

Figure S9B. HPLC profile of C2 and C5 compounds in comparison with culture filtrate extract.

Table S1. Composition of different media used in this study (Above mentioned all media without agar served as broth media)

SL No.	Name of Media	Media Composition
**1**	SOC	Tryptone- 20 g/L<br>Yeast extract- 5 g/L<br>NaCl- 0.5 g/L<br>KCl (1 M solution)- 2.5 mL<br>Glucose (1 M solution)- 20 mL after sterilization<br>$MgCl_2$ (2 M solution)- 5 mL after sterilization<br>pH 7.0
**2**	NZY	NaCl- 5 g/L<br>$MgSO_4 \times 7H\ 2O$- 2 g/L<br>Yeast extract 5 g/L<br>NZ – amine 10 g/L
**3**	MEX-A	Biomalt extract 20 g/L<br>Yeast extract 0.1 g/L,<br>Glycerine 50 g/L
**4**	SPG	Soya peptone 10 g/L<br>Glucose 4 g/L<br>KH_2PO_4 1 g/L
**5**	Potato Dextrose Yeast extract Broth (PDB)	Potato infusion 200g<br>Dextrose 20g<br>Yeast extract 3g
**6**	Modified medium -1- Agar(M1DA)	Sucrose- 30 g/L<br>Ammonium tartrate- 5g/L<br>Yeast extract - 0.5 g/L<br>Soytone - 1g/L<br>$Ca_2 (NO)_3$ - 280 mg/L<br>KNO_3 - 80 mg/L<br>KCl- 60 mg/L<br>$MgSO_4$ - 360 mg/L<br>NaH_2PO_4 - 20 mg/L<br>H_3BO_3 1.4 mg/L<br>$MnSO_4$ 5 mg/L<br>$ZnSO_4$ 2.5 mg/L<br>KI 0.7 mg and Agar 20 mg/L
**7**	Malt extract agar	Malt extract - 20 g/L<br>Agar- 20 g/L
**8**	S-7 AGAR	Glucose- 1 g/L<br>Fructose- 3 g/L<br>Sucrose- 6 g/L<br>Na+ acetate- 1 g/L<br>Soytone- 1 mg/L<br>Thiamine-1 mg/L<br>Biotin- 1mg/L<br>Pyridoxal-1 mg/L<br>Ca_2+ pantothenate- 1 mg/L<br>$MgSO_4$ - 2.5 mg /L<br>$MnCl_2$ 5 mg/L<br>$FeCl_2$ 2 mg/L<br>phenylalanine 5 mg/L<br>Na+ benzoate 100 mg/L
**9**	Antibiotic Production medium –II (AP-II)	Soluble starch- 2.5g/L<br>Glucose- 10.0g/L<br>Yeast extract- 2g/L<br>$CaCO_3$- 3g/L<br>**Trace salt Solution**- 1ml<br>$FeSO_4 \cdot 7H\ 2O$ 0.1 g/L<br>$MnCl_2$ 0.1 g/L<br>$ZnSO_4 \cdot 7H\ 2O$ 0.1 g/L<br>pH: 7.2
**10**	Malt-Yeast Extract Medium	Yeast extract- 4 g/L<br>Malt extract- 10 g/L<br>Glucose-4 g/L<br>Agar 15 g/L

**11**	Potato Dextrose Yeast Extract Broth (PDYEB)	200 g of potato infusion, 20g dextrose<br>3 g Yeast extract

Table S2. Species richness and diversity indices of marine endophytic fungi obtained from Green, brown and red algae.

Type of algae	Total species occurrence	Mean species richness	Shannon index	Simpson's index	Menhinick's index
**Green algae**	47	5.22	3	15.74	4.23
**Brown algae**	25	5	2.5	9.45	4
**Red algae**	21	6	2.58	9.96	4.08

Table S3. Cytotoxicity and IC_{50} value of marine endophytic fungal extract against two cancer cell lines HeLa and A431.

S. No.	Fungal strains	Normal HEK cells CC_{50} value µg/ml	Cancer cells CC_{50} value µg/ml		Selectivity index (SI)	
			**A431**	**HeLa**	**A431**	**HeLa**
1	*Aspergillus tamarii*	1008.55	63.01	105.75	16.00	9.53
2	*A. terrus*	1035.92	62.57	54.24	16.55	19.09
3	*Amesia atrobrunnea*	663.60	55.43	82.14	11.97	8.07
4	*A. amstelodami*	609.10	42.17	84.25	14.44	7.22
5	*Periconia byssoides*	456.56	26.01	17.96	17.55	25.42
6	*A.tubingensis*	2313.38	165.53	96.82	13.97	23.89
7	*A.niger*	247.476	69.26	48.97	3.57	5.05
8	*Phoma moricola*	437.93	57.77	40.56	7.58	10.79
9	*A.amstelodami*	229.00	93.21	55.71	2.45	4.11
10	*Cladosporium tenuissimum*	254.71	42.62	37.48	5.97	6.79
11	*Choniochaeta* sp.	578.95	28.55	18.29	20.27	31.65
12	*Hortaea werneckii*	550.23	60.78	46.71	9.05	11.77
13	*Amesia atrobrunneea*	5319.72	53.66	62.80	99.13	84.70
14	*Trichoderma erinaceum*	–	76.46	46.89	NA	NA
15	*Aplosporella artocarpi*	–	102.46	47.89	NA	NA
16	*Alternaria alternata*	3433.89	33.97	60.38	101.08	56.87
17	*C. cladosporoides*	459.79	28.54	18.92	16.11	24.30
18	*C. tenuissimum*	477.38	39.78	37.55	12.00	12.71
19	*A. ochraceopetaliformis*	213.73	37.38	42.70	5.71	5.00
20	*Aplosporella artocarpi*	–	45.12	50.76	NA	NA
21	*Biscogniauxia petrensis*	–	24.85	18.04	NA	NA
22	*Nigrospora oryzae*	–	25.64	28.49	NA	NA
23	*Gymnascella aurantiaca*	–	25.47	18.88	NA	NA
24	*Gliomastix murorum*	664.624	30.27	18.22	21.95	36.47
25	*Nectria dematicosa*	848.374	34.59	18.04	24.52	47.02
26	*Ascotricha sinuosa*	665.989	33.42	28.38	19.92	23.46
27	*Daldinia eschscholtzii*	236.734	112.51	61.51	2.10	3.84
28	*C. xantochromaticum*	218.999	66.85	70.62	3.27	3.10
29	*Ascotricha chartarum*	167.140	49.22	105.75	3.39	1.58
30	*A. amoenus*	874.671	100.39	54.24	8.71	16.12
31	*Periconia celaeidis*	203.386	45.28	82.14	4.49	2.47

CC_{50}: 50% cytotoxic concentration, selective index (SI) = CC_{50} of cancer cells /CC_{50} of Normal healthy cells (HEK), – No activity and NA-Not applicable.

Table S4A. TLC band character of mycelia extract of *Biscogniauxia petrensis*.

**S. No.**	**Spot**	**R*f***	**Colour**		
			**Visible**	**UV λ_{254}**	**UV λ_{365}**
**1**	M1	0.82	−	Light blue	Dark blue
**2**	M2	0.77	−	Black	Light Blue
**3**	M3	0.71	−	Black	Violet blue
**4**	M4	0.66	Light brown	Light Black	Reddish orange
**5**	M5	0.61	−	−	Violet
**6**	M6	0.56	−	−	Light Violet
**7**	M7	0.46	−	−	Blue
**8**	M8	0.39	−	−	Light Blue
**9**	M9	0.32	−	−	Light Blue
**10**	M10	0.23	−	−	Light Blue
**11**	M11	0.018	−	−	Light Blue
**12**	M12	0.015	−	−	Light Blue
**13**	M13	0.12	Light brown	−	Light orange
**14**	M14	0.10	Light brown	−	Blue
**15**	M15- Origin	0.07	−	−	Yellowish

− Not visible

Table S4B. TLC band character of culture filtrate extract of *Biscogniauxia petrensis*.

S. No.	Spot	Rf value	Colour		
			**Visible**	**UV λ_{254}**	**UV λ_{365}**
**1**	C1	0.96	−	Blue	Dark blue
**2**	C2	0.88	−		Orange
**3**	C3	0.81	−	Blue	Light orange
**4**	C4	0.76	−	Blue	Reddish orange
**5**	C5	0.70	Light yellow	Light blue	Dark Yellow
**6**	C6	0.61	−		Light yellow
**7**	C7	0.58	−	Light blue	Brown
**8**	C8	0.50	−		Dark Yellow
**9**	C9	0.46	Light brown	Blue	Light Brown
**10**	C10	0.41	Light brown	Blue	Green
**11**	C11	0.33	−	−	Light green
**12**	C12	0.27	−	−	Violet
**13**	C13	0.23	−	−	Light violet
**14**	C14	0.20	−	−	Dark Yellow
**15**	C15	0.15	Light pink	Blue	Light Yellow
**16**	C16	0.10	Dark pink	Blue	Violet (Thick)
**17**	C17	0.08			Dark Yellow
**18**	C18	0.03	Yellow	Dark blue	Light yellow
**19**	C19- Origin	0.03	Thick brown	Black	Dark brown

− Not visible

Table S5. The putative identifications of bioactive compounds compared with direct Mass-based search selected peaks in MetFrag data base.

S. No.	Fungal fraction	MetFrag Identifier	RT (mins)	Mass of Ion	Base peak (MS)	Fragments of ion (MS/MS)	Compound Formula	Compound Name
**1**	C2	20790	32.1	212.0202	193.0790<br>167.9944	194.0095<br>167.9946<br>136.0217	$C_9H_9NOS_2$	2-(1,3-benzothiazol-2-ylsulfanyl)ethanol
**2**	C5	88701048	12.3	185.1152	107.0592<br>128.0529	147.0586<br>181.0381<br>165.0419	$C_8H_{14}N_3O_2$	5-cyclohexyl-1-oxido-triazolidin-4-one
**3**	M3	59994049	27.9	229.0834	170.0592<br>189.0908<br>207.1015	189.0904<br>161.0958<br>151.5358	$C_{14}H_{12}O_3$	3-hydroxy-7-propyl-naphthalene-2-carboxylic acid
**4**	M4	14552494	13.5	185.1153	179.0231<br>207.0997	128.5173<br>137.5214<br>147.0325	$C_6H_{12}N_6O$	2,2-bis(azidomethyl)butan-1-ol
